# Non-cigarette tobacco cessation support in England: current provision, confidence, and barriers identified in a cross-sectional survey of healthcare professionals involved in smoking cessation support

**DOI:** 10.1093/ntr/ntag071

**Published:** 2026-04-03

**Authors:** Yo Seop Byun, Eve Taylor, Lion Shahab, Katherine East, Jamie Brown, Harry Tattan-Birch, Sharon Cox, Masuma Pervin Mishu, David Hammond, Sarah E Jackson

**Affiliations:** Department of Behavioural Science and Health, University College London, London WC1E 6BT, United Kingdom; Department of Behavioural Science and Health, University College London, London WC1E 6BT, United Kingdom; Department of Behavioural Science and Health, University College London, London WC1E 6BT, United Kingdom; Department of Primary Care and Public Health, Brighton and Sussex Medical School, Brighton BN1 9PX, United Kingdom; National Addiction Centre, Institute of Psychiatry, Psychology & Neuroscience, Kings College London, London SE5 8AF, United Kingdom; Department of Behavioural Science and Health, University College London, London WC1E 6BT, United Kingdom; Behavioural Research UK, Edinburgh, United Kingdom; Department of Behavioural Science and Health, University College London, London WC1E 6BT, United Kingdom; Department of Behavioural Science and Health, University College London, London WC1E 6BT, United Kingdom; Behavioural Research UK, Edinburgh, United Kingdom; Department of Epidemiology and Public Health, University College London, London WC1E 6BT, United Kingdom; School of Public Health Sciences, University of Waterloo, Waterloo, Ontario N2L 3G1, Canada; Department of Behavioural Science and Health, University College London, London WC1E 6BT, United Kingdom

**Keywords:** non-cigarette tobacco, cigars, shisha, chewing tobacco, smokeless tobacco, heated tobacco, cessation support, stop smoking services, harm perceptions

## Abstract

**Introduction:**

While cigarette smoking remains the dominant form of tobacco use in England, a range of other tobacco products are also used. Evidence on demand for non-cigarette tobacco cessation support—and on providers’ preparedness and barriers to offering such support—remains limited.

**Methods:**

We conducted a cross-sectional online survey of professionals involved in smoking cessation support in England (*n* = 96). For each specific non-cigarette tobacco product (cigars, cigarillos, shisha, pipes, bidis, chewing tobacco, dry snuff, moist snuff/dip, and heated tobacco), the survey assessed awareness, perceived harms, demand for cessation support, interventions offered, training and confidence, and perceived barriers to providing support.

**Results:**

Awareness was universal for cigars, pipes, and shisha; high for chewing tobacco (87.5%), heated tobacco (77.1%), and cigarillos (71.9%); and lower for dry snuff (56.3%), bidis (33.3%), and moist snuff/dip (26.0%). Client demand for cessation support was low: 20.8% of participants reported requests at least monthly for cigars and ≤13.5% for other products. Support was mainly behavioral and nicotine replacement therapy-based, with few (<25%) offering other pharmacotherapies. Most participants who offered support used the same approach as for cigarettes (>90% across products), with few having received product-specific training (2.1%–19.8% of all participants). The main barriers included perceived lack of demand (46.9%), training (44.8%), and knowledge (43.8%); open-ended responses also emphasized that commissioning constraints excluded non-cigarette products.

**Conclusions:**

Healthcare professionals in England report limited demand for cessation support for non-cigarette tobacco products, which may partly reflect service reach and commissioning constraints rather than lack of need. Few report receiving product-specific training; most apply standard cigarette cessation approaches or are not commissioned to provide support.

**Implications:**

Healthcare professionals involved in smoking cessation support in England often lack the training, knowledge, and funding to support people to quit non-cigarette tobacco. Including these products in service plans and commissioning would enable them to give proper support.

## Introduction

While cigarette smoking remains the most common form of tobacco use in England,^1^ a range of other smoked and smokeless tobacco products are also used.^2^ These products differ substantially in form, consumption patterns, nicotine delivery, and health risks—ranging from combustible products with high toxicant exposure to non-combustible options with lower but still significant risks.^3–7^ Importantly, all non-cigarette tobacco products remain more harmful than abstaining from tobacco use entirely.^3–7^ Yet evidence on the demand for cessation support for these products—and on healthcare professionals’ preparedness and perceived barriers to offering such support—remains limited. Existing tobacco-dependence treatment systems, including local-authority funded stop smoking services which are widely available in England and free at the point of access, were developed primarily to support cigarette cessation, raising questions about their capacity to meet changing patterns of tobacco use and dependence.

Recent evidence suggests that non-cigarette tobacco use is an important and evolving component of England’s tobacco landscape. Approximately 3.7% of adults—around 1.7 million people—report currently using at least one non-cigarette tobacco product, with cigars and shisha currently the most commonly used.^2^ Use is socially patterned and often shaped by cultural norms: for example, shisha is more frequently used within Middle Eastern communities,^8–10^ and bidis and chewing tobacco have established use among South Asian populations.^11–15^ Recent population data show that cigar, cigarillo, and pipe use is more common among men and those who also smoke cigarettes, whereas shisha use is concentrated among younger adults, minority ethnic communities, and people who vape.^2^ While smokeless tobacco is disproportionately used by people who also smoke tobacco, some people only use smokeless products.^2^

Evidence suggests non-cigarette tobacco use in England has grown in recent years. According to the Smoking Toolkit Study, the proportion of adults who do not smoke cigarettes at all but smoke some other form of tobacco increased from 0.36% in 2013 to 1.8% in 2023 (~772 800 people), with the steepest increases occurring among young adults since 2020.^16^ Similar patterns are evident among youth. According to the ITC Youth Tobacco and Vaping Survey, the prevalence of any non-cigarette tobacco use among 16–19-year-olds in England increased from 7.4% in 2017 to 11.6% in 2024, in contrast to declining use in Canada and the United States over the same period.^17^ Much of this rise was driven by increasing cigarillo, cigar, and smokeless tobacco use.^17^ These trends are consistent with HM Revenue and Customs data showing that UK cigar sales increased from £73 million in 2019 to £165 million in 2024, with clearances figures following a similar pattern.^18^

Together, these findings show that non-cigarette tobacco is now used by substantial numbers of adults and youth in England, yet current cessation support systems were designed for cigarettes.^19^ Guidelines recommend that healthcare professionals routinely assess smokeless tobacco use, advise users on health risks, and offer or refer for cessation support.^14^ However, in practice, these recommendations may not be consistently implemented, and some healthcare professionals who provide smoking cessation support may have limited training, confidence, or resources to support users of smokeless or other non-cigarette tobacco products. It therefore remains unclear whether existing services can adequately meet the needs of people using alternative tobacco products. This study addresses these gaps by examining healthcare professionals’ awareness, confidence, training, and current practices related to non-cigarette tobacco cessation across England. Specifically, the study aimed to address the following research questions:

1. Which non-cigarette tobacco products do healthcare professionals involved in smoking cessation support encounter people seeking support to quit?
2. How confident are they in supporting people to stop using these products?
3. Have they received any training in this?
4. What support do they currently offer?
5. What are the main barriers to offering support?

## Methods

### Design and sample

Data were collected via a cross-sectional online survey using the REDCap online survey platform between June 25 and August 7, 2024. Eligible participants were healthcare professionals aged ≥18 years, based in England, and involved in providing cessation support (eg, stop smoking service practitioners and advisors in primary care and local authority-commissioned services). Recruitment occurred via emails targeting stop smoking services (distributed by research team members, the National Centre for Smoking Cessation and Training [NCSCT], and other tobacco control contacts) and social media advertisements (LinkedIn). Participants could optionally provide contact details to enter a prize draw for one of six £50 vouchers.

A total of 132 people responded, of whom 96 (72.2%) were eligible for inclusion and completed the survey, forming the analytic sample (of those excluded, one was not based in England and the remainder did not complete the survey, with most dropping out before completing the initial section assessing participant characteristics). All participants were screened for implausibly fast response times and duplicate email addresses to identify potential automated responses; no evidence of such issues was found. Ethical approval was obtained from the UCL Research Ethics Committee (ID 0780); all participants provided written informed consent before completing the survey.

### Measures

Data were collected using a structured questionnaire co-developed with patient and public involvement representatives and tobacco cessation experts. The questionnaire items are provided in full in Supplementary File 1. The questionnaire captured four main domains:

1. Participant characteristics, including job role, workplace setting, region, years of experience, and service focus.
2. Awareness and perceptions of non-cigarette tobacco products. Participants were asked about nine products—cigars, cigarillos, pipes, shisha, heated tobacco, chewing tobacco, bidis, dry snuff, and moist snuff/dip. Visual examples and brief descriptions accompanied each product to facilitate accurate identification. For each product participants were aware of, they were asked how harmful they perceived the product to be relative to cigarettes and whether the product causes cancer.
3. Confidence, training, and support provision. For each product participants were aware of, the questionnaire assessed whether they had received product-specific training, their confidence in providing cessation support, whether their service offered support for that product, and perceived service demand. Participants also indicated the types of interventions provided, including behavioral support, nicotine replacement therapy (NRT), varenicline, cytisine, bupropion, e-cigarettes, nicotine pouches, referral to other support, or other interventions. Open-text responses captured types of training received and ways in which they made adaptations relative to cigarette cessation support.
4. Barriers and resource needs. All participants were asked to select from a list of potential barriers to providing support for non-cigarette tobacco cessation. Open-text responses captured other barriers to supporting cessation and desired resources to improve support provision.

### Statistical analysis

All analyses were conducted using SPSS version 29. The analyses were not pre-registered and should be considered exploratory.

Participant characteristics were summarized using frequencies and percentages for categorical variables and medians and interquartile ranges for continuous variables. Responses related to non-cigarette tobacco cessation service demand, beliefs about harms and health risks, training, confidence, provision of support, and differences between smoking and non-cigarette tobacco cessation approaches were summarized using frequencies and proportions. Open-ended responses regarding training received, types of support offered, differences in support, barriers, and resources were coded inductively using descriptive content analysis to identify recurring ideas, which were summarized narratively. Raw open-ended responses are provided in Supplementary File 2.

## Results

### Sample characteristics

We analyzed responses from 96 participants involved in providing smoking cessation support, representing all regions of England (Table 1). The majority (88.5%; *n* = 85) were specialist stop smoking or tobacco dependence advisors, with the remainder including a general practitioner and professionals in management, coordination, clinical, or pharmacy roles. Over half of participants (56.3%) worked in community settings, and 42.7% delivered services targeting specific populations, most commonly pregnant individuals, people with mental health conditions, ethnic minority groups, young people, and those living in deprived areas. Additional groups supported included individuals with learning disabilities, autism, and people with substance dependencies and people experiencing homelessness. Most services (61.5%) had eligibility criteria for access, such as being a current smoker (rather than exclusively vaping), meeting minimum age thresholds (usually 12 or 18 years), residing, working, or being registered with a general practitioner (GP) in the service area, or belonging to a priority group (eg, pregnant or postnatal clients, inpatients, or people with mental health conditions). Participants reported a median of 4 years’ experience in smoking cessation support (range <1–26 years). Most (76.0%) indicated that clients sought support for smoking cessation at least daily, and for the majority (67.7%), the typical duration of smoking cessation support offered to clients was between 9 and 12 weeks.

**Table 1:** Sample characteristics.

Characteristic	% (*n*)^a^
**Professional role**	
** Stop smoking/tobacco dependency advisor**	88.5 (85)
** Service manager/lead**	5.2 (5)
** Coordinator/advisor in local authority public health team**	2.1 (2)
** Clinical staff**	2.1 (2)
** General practitioner**	1.0 (1)
** Pharmacy support staff**	1.0 (1)
	
**Primary workplace setting**	
** Community**	56.3 (54)
** Hospital—inpatient**	20.8 (20)
** Hospital—outpatient**	4.2 (4)
** Primary care**	2.1 (2)
** Remote (eg, online, app, telephone)**	15.6 (15)
** Other (eg, multiple settings)**	3.1 (3)
	
**Service focuses on a specific population**	42.7 (41)
**Service has eligibility criteria for cessation support**	61.5 (59)
	
**How often clients approach service for smoking cessation support**	
** Daily**	76.0 (73)
** Less than daily but at least weekly**	17.7 (17)
** Less than weekly but at least monthly**	6.3 (6)
	
**Typical duration of support provided**	
** 4–8 weeks**	6.3 (6)
** 9–12 weeks**	67.7 (65)
** >12 weeks**	26.0 (25)
	
**Region of England**	
** North East**	9.4 (9)
** North West**	7.3 (7)
** Yorkshire and the Humber**	5.2 (5)
** West Midlands**	17.7 (17)
** East Midlands**	25.0 (24)
** East of England**	3.1 (3)
** London**	8.3 (8)
** South East**	11.5 (11)
** South West**	2.1 (2)
** All of England**	10.4 (10)
	
**Ethnic group**	
** White**	79.2 (76)
** Asian**	16.7 (16)
** Black**	4.2 (4)
	
**How long provided smoking cessation support (years), median (IQR); min-max**	4 (8); <1–26

^a^Unless otherwise specified. IQR, interquartile range. Min, minimum. Max, maximum.

### Non-cigarette tobacco product awareness, perceived harm, and health risks

Table 2 summarizes awareness and perceptions of non-cigarette tobacco products. Awareness was universal for cigars, shisha, and pipes (all 100%), and remained high for chewing tobacco (87.5%), heated tobacco (77.1%), and cigarillos (71.9%). In contrast, awareness was lower for dry snuff (56.3%), with fewer than half of participants aware of bidis (33.3%) or moist snuff/dip (26.0%).

**Table 2:** Non-cigarette tobacco product awareness, perceived harm, and health risks.

	% (*n*)								
Measure	Cigars	Cigarillos	Shisha	Pipes	Bidis	Chewing tobacco	Dry snuff	Moist snuff/dip	Heated tobacco
**Aware of product** ^**a**^									
** Yes**	100 (96)	71.9 (69)	100 (96)	100 (96)	33.3 (32)	87.5 (84)	56.3 (54)	26.0 (25)	77.1 (74)
** No**	0 (0)	25.0 (24)	0 (0)	0 (0)	65.6 (63)	12.5 (12)	42.7 (41)	70.8 (68)	20.8 (20)
** Don’t know**	0 (0)	3.1 (3)	0 (0)	0 (0)	1.0 (1)	0 (0)	1.0 (1)	3.1 (3)	2.1 (2)
									
**Perceived harm compared with cigarettes** ^**b**^									
** Not harmful at all**	0 (0)	0 (0)	0 (0)	0 (0)	0 (0)	0 (0)	0 (0)	0 (0)	1.4 (1)
** A lot less harmful**	0 (0)	0 (0)	3.1 (3)	0 (0)	0 (0)	4.8 (4)	5.6 (3)	4.0 (1)	4.1 (3)
** A little less harmful**	2.1 (2)	4.3 (3)	12.5 (12)	4.2 (4)	6.3 (2)	15.5 (13)	35.2 (19)	32.0 (8)	48.6 (36)
** About the same**	54.2 (52)	59.4 (41)	41.7 (40)	67.7 (65)	62.5 (20)	36.9 (31)	27.8 (15)	40.0 (10)	36.5 (27)
** A little more harmful**	18.8 (18)	21.7 (15)	11.5 (11)	18.8 (18)	6.3 (2)	17.9 (15)	9.3 (5)	8.0 (2)	5.4 (4)
** A lot more harmful**	25.0 (24)	8.7 (6)	25.0 (24)	8.3 (8)	25.0 (8)	19.0 (16)	3.7 (2)	4.0 (1)	0 (0)
** Don’t know**	0 (0)	5.8 (4)	6.3 (6)	1.0 (1)	0 (0)	6.0 (5)	18.5 (10)	12.0 (3)	4.1 (3)
									
**Believe that product causes cancer** ^**b**^									
** Yes**	100 (96)	98.6 (68)	84.4 (81)	96.9 (93)	96.9 (31)	92.9 (78)	66.7 (36)	72.0 (18)	75.7 (56)
** No**	0 (0)	0 (0)	2.1 (2)	0 (0)	0 (0)	1.2 (1)	3.7 (2)	12.0 (3)	4.1 (3)
** Don’t know**	0 (0)	1.4 (1)	13.5 (13)	3.1 (3)	3.1 (1)	6.0 (5)	29.6 (16)	16.0 (4)	20.3 (15)

^a^Among all participants (*n* = 96).

^b^Among participants aware of the product.

Among participants who were aware of each product, perceived harm compared with cigarettes varied across products. Of the smoked products, most participants viewed cigars, cigarillos, pipes, and bidis as about as harmful as cigarettes (54.2%–67.7%). Perceptions of shisha were more uncertain, with a smaller proportion (41.7%) rating it as about as harmful as cigarettes. For the smokeless products, perceived harm was generally lower than for smoked products, with fewer participants perceiving them as equally harmful: chewing tobacco (36.9%), dry snuff (27.8%), moist snuff/dip (40.0%), and heated tobacco (36.5%). Very few participants considered any product to be not harmful at all.

Beliefs about cancer risk followed a similar pattern. For smoked products, almost all participants believed cigars (100%), cigarillos (98.6%), bidis (96.9%), and pipes (96.9%) cause cancer, with a slightly lower proportion for shisha (84.4%). For smokeless products, endorsement was generally lower: while almost all participants believed chewing tobacco (92.9%) causes cancer, fewer believed this for heated tobacco (75.7%), dry snuff (66.7%) or moist snuff/dip (72.0%). Uncertainty about cancer risk was common for these latter two products (29.6% and 16.0%, respectively).

### Demand for and provision of non-cigarette tobacco cessation support

Table 3 presents findings on demand for and provision of cessation support, among all participants surveyed (those not aware of the product were treated as neither seeing clients seeking support, nor offering support, for product cessation). Across all products, few participants reported clients seeking cessation support frequently. Requests were most common for cigars (at least monthly: 20.8%; at least yearly: 50.0%), heated tobacco (13.5%; 31.1%), and shisha (11.5%; 34.4%) and least common for moist snuff/dip (1.0%; 2.1%) and dry snuff (0%; 2.1%).

**Table 3:** Demand for and provision of non-cigarette tobacco cessation support.

	% (*n*)								
Measure	Cigars	Cigarillos	Shisha	Pipes	Bidis	Chewing tobacco	Dry snuff	Moist snuff/dip	Heated tobacco
**Frequency of clients seeking support for product cessation** ^**a**^									
** At least weekly**	6.3 (6)	2.1 (2)	3.1 (3)	0 (0)	1.0 (1)	0 (0)	0 (0)	0 (0)	0 (0)
** At least monthly**	20.8 (20)	9.4 (9)	11.5 (11)	4.2 (4)	5.2 (5)	6.3 (6)	0 (0)	1.0 (1)	13.5 (13)
** At least yearly**	50.0 (48)	29.2 (28)	34.4 (33)	25.0 (24)	9.4 (9)	19.8 (19)	2.1 (2)	2.1 (2)	31.3 (30)
									
**Offer support for product cessation** ^**a**^									
** Yes**	82.3 (79)	55.2 (53)	54.2 (52)	68.8 (66)	22.9 (22)	40.6 (39)	11.5 (11)	9.4 (9)	37.5 (36)
** No**	11.5 (11)	36.5 (35)	25.0 (24)	16.7 (16)	75.0 (72)	37.5 (36)	63.5 (61)	84.4 (81)	46.9 (45)
** Don’t know**	6.3 (6)	8.3 (8)	20.8 (20)	14.6 (14)	2.1 (2)	21.9 (21)	25.0 (24)	6.3 (6)	15.6 (15)
									
**Types of support offered for product cessation^a,b^**									
** Behavioral support**	78.1 (75)	52.1 (50)	50.0 (48)	66.7 (64)	22.9 (22)	39.6 (38)	11.5 (11)	9.4 (9)	37.5 (36)
** Nicotine replacement therapy**	82.3 (79)	55.2 (53)	52.1 (50)	66.7 (64)	20.8 (20)	38.5 (37)	9.4 (9)	7.3 (7)	35.4 (34)
** Varenicline**	25.0 (24)	14.6 (14)	14.6 (14)	17.7 (17)	6.3 (6)	9.4 (9)	3.1 (3)	1.0 (1)	10.4 (10)
** Cytisine**	19.8 (19)	12.5 (12)	9.4 (9)	12.5 (12)	5.2 (5)	8.3 (8)	3.1 (3)	1.0 (1)	10.4 (10)
** Bupropion**	19.8 (19)	15.6 (15)	8.3 (8)	16.7 (16)	6.3 (6)	7.3 (7)	4.2 (4)	2.1 (2)	9.4 (9)
** Provision of e-cigarettes**	51.0 (49)	38.5 (37)	33.3 (32)	43.8 (42)	15.6 (15)	25.0 (24)	8.3 (8)	4.2 (4)	27.1 (26)
** Advice on e-cigarettes**	52.1 (50)	35.4 (34)	31.3 (30)	45.8 (44)	14.6 (14)	21.9 (21)	8.3 (8)	5.2 (5)	26.0 (25)
** Advice on nicotine pouches**	11.5 (11)	9.4 (9)	9.4 (9)	11.5 (11)	5.2 (5)	9.4 (9)	4.2 (4)	3.1 (3)	7.3 (7)
** Referral to other support**	14.6 (14)	7.3 (7)	5.2 (5)	10.4 (10)	6.3 (6)	6.3 (6)	3.1 (3)	3.1 (3)	6.3 (6)
** Other**	2.1 (2)	1.0 (1)	0 (0)	1.0 (1)	1.0 (1)	0 (0)	0 (0)	0 (0)	0 (0)
** None of these**	17.7 (17)	44.8 (43)	45.8 (44)	31.3 (30)	77.1 (74)	59.4 (57)	88.5 (85)	90.6 (87)	62.5 (60)
									
**Support differs from cigarette cessation** ^**c**^									
** Yes**	2.5 (2)	3.8 (2)	5.8 (3)	3.0 (2)	0 (0)	12.8 (5)	18.2 (2)	11.1 (1)	5.6 (2)
** No**	93.7 (74)	96.2 (51)	84.6 (44)	93.9 (62)	90.9 (20)	76.9 (30)	81.8 (9)	88.9 (8)	94.4 (34)
** Don’t know**	3.8 (3)	0 (0)	9.6 (5)	3.0 (2)	9.1 (2)	10.3 (4)	0 (0)	0 (0)	0 (0)

^a^Among all participants (*n* = 96). Those not aware of the product were treated as neither seeing clients seeking support, nor offering support, for product cessation.

^b^Not mutually exclusive; participants were asked to select all that applied.

^c^Among participants who reported providing support for product cessation; valid percentages shown.

Provision of support for each product generally reflected patterns of demand, with participants more likely to offer support for smoked than smokeless products. Most participants reported offering cessation support for cigars (82.3%), with moderate levels for pipes (68.8%), cigarillos (55.2%), and shisha (54.2%). Support was less common for chewing tobacco (40.6%) and heated tobacco (37.5%), and rare for bidis (22.9%), dry snuff (11.5%), and moist snuff/dip (9.4%).

Behavioral support and NRT were the most commonly reported interventions across all products, with particularly high rates of provision for cigars (78.1% and 82.3%, respectively) and pipes (66.7% for both). Advice or provision of e-cigarettes was also reported by some (eg, 52.1% and 51.0% for cigars, respectively). Pharmacotherapies such as varenicline, cytisine, and bupropion were much less frequently offered (<25% across all products). Very few participants offered advice on nicotine pouches (3.1%–11.5% across products) or referrals to other support (3.1%–14.6%).

### Differentiated approaches to non-cigarette tobacco cessation support

Most participants who provided support indicated that their approach to non-cigarette product cessation did not differ from support for stopping smoking cigarettes, particularly for cigars, cigarillos, pipes, and bidis (all >90%; Table 3). However, open-ended responses highlighted several ways in which support was tailored to specific products (Supplementary File 2).

Adaptations to behavioral support were the most commonly cited modifications in the open-ended responses. Participants emphasized the importance of understanding unique usage patterns and product-specific barriers, including social or cultural factors. One participant noted: “We’d discuss how and why they use cigars and the barriers to quitting. The association with cigars and it not being tobacco and the use in celebrations etc.” Similar considerations were reported for cigarillos, pipes, and shisha, with attention to frequency, context, and social patterns: “Normally these people are not doing it every day but occasionally and socially,” and “we would talk about when, how and how often. Whether there is a social aspect, e.g., at shisha café, etc.”

Treatment limitations were also frequently noted, reflecting service restrictions and commissioning frameworks. For smokeless products such as chewing tobacco, dry snuff, and moist snuff/dip, many participants reported that support was restricted to behavioral approaches, with limited access to pharmacotherapy. One participant explained: “We as a stop smoking service are NOT commissioned to provide support for chewing tobacco users, only combustible tobacco. However, due to the population demand, we do offer behavioral support and NRT for these service users. The NRT gum is the most preferred product of service user choice as it replicates the chewing tobacco sensation.” Restrictions on treatments for shisha were also reported: “We do not offer NRT/Vapes for shisha use. We would offer behavioural support for users.”

Product-specific harm and risk considerations were incorporated into discussions with clients. Participants highlighted differences in carbon monoxide (CO) exposure, tar, and oral versus respiratory cancers. For example, one participant described providing “relevant info on dangers of shisha,” while another noted that support for cigarillos was “similar but [had] more emphasis on CO gas and tar.”

### Training and confidence in providing non-cigarette tobacco cessation support

Table 4 summarizes training and confidence among all participants. Overall, relatively few participants reported having received product-specific cessation training. Training was most common for shisha (19.8%), followed by cigars (17.7%), pipes (16.7%), heated tobacco (14.6%), and chewing tobacco (13.5%). For all other products, receipt of training was less common (<10%).

**Table 4:** Training and confidence.

	% (*n*)								
Measure	Cigars	Cigarillos	Shisha	Pipes	Bidis	Chewing tobacco	Dry snuff	Moist snuff/dip	Heated tobacco
**Received training in product cessation** ^**a**^									
** Yes**	17.7 (17)	9.4 (9)	19.8 (19)	16.7 (16)	8.3 (8)	13.5 (13)	4.2 (4)	2.1 (2)	14.6 (14)
** No**	74.0 (71)	84.4 (81)	75.0 (72)	75.0 (72)	89.6 (86)	77.1 (74)	92.7 (89)	94.8 (91)	83.3 (80)
** Don’t know**	8.3 (8)	6.3 (6)	5.2 (5)	8.3 (8)	2.1 (2)	9.4 (9)	3.1 (3)	3.1 (3)	2.1 (2)
									
**Confidence in providing support for product cessation** ^**b**^									
** Not at all confident**	1.0 (1)	1.4 (1)	8.3 (8)	1.0 (1)	0 (0)	14.3 (12)	33.3 (18)	24.0 (6)	4.1 (3)
** Not very confident**	8.3 (8)	5.8 (4)	30.2 (29)	22.9 (22)	28.1 (9)	34.5 (29)	35.2 (19)	32.0 (8)	23.0 (17)
** Somewhat confident**	46.9 (45)	47.8 (33)	40.6 (39)	50.0 (48)	28.1 (9)	32.1 (27)	25.9 (14)	36.0 (9)	41.9 (31)
** Very confident**	38.5 (37)	37.7 (26)	17.7 (17)	22.9 (22)	37.5 (12)	13.1 (11)	3.7 (2)	8.0 (2)	23.0 (17)
** Extremely confident**	5.2 (5)	7.2 (5)	2.1 (2)	3.1 (3)	6.3 (2)	2.4 (2)	0 (0)	0 (0)	2.7 (2)
** Don’t know**	0 (0)	0 (0)	1.0 (1)	0 (0)	0 (0)	3.6 (3)	1.9 (1)	0 (0)	5.4 (4)

^a^Among all participants (*n* = 96). Those not aware of the product treated as not having received training.

^b^Among participants aware of the product (*n* per product shown in Table 1).

Among those who had received training, the NCSCT was the most frequently cited source in open-ended responses, followed by local or service-specific programs (Supplementary File 2). For cigars and cigarillos, participants often reported that the training they received was the same as for cigarettes, indicating a largely generic rather than product-specific focus. One participant explained: “I apply the same training as I received to help people quit cigarettes. I don’t make a distinction between cigarettes and cigars; they both contain tobacco and cause nicotine addiction.” For less commonly used products—such as heated tobacco, bidis, and dry snuff—some participants described receiving only basic information on what the products are and how they work. In contrast, training for culturally specific products, including shisha, bidis, and chewing tobacco, sometimes incorporated a demographic or cultural focus, reflecting their higher prevalence within certain communities. One participant highlighted this approach, stating: “we had a workshop on different smoking habits and products that were more common in some Asian communities,” while another described shisha training as including “information on use amongst different demographic groups, how it’s used and [its] impact.”

Among participants who were aware of each product, confidence in providing cessation support varied substantially. Overall, participants reported higher confidence for smoked products than for smokeless products. Confidence was highest for cigars, cigarillos, and bidis (around 44% very or extremely confident) but lower for pipes (26.0%) and shisha (19.8%). Among smokeless products, confidence was highest for heated tobacco (25.7%), followed by chewing tobacco (15.5%), moist snuff/dip (8.0%), and dry snuff (3.7%).

### Barriers to providing non-cigarette tobacco cessation support

Table 5 summarizes reported barriers. The most frequently endorsed barriers to providing cessation support were lack of demand (46.9%), lack of training (44.8%), and lack of knowledge (43.8%). Other barriers cited by a notable minority of participants included lack of confidence (20.8%), language barriers (19.8%), and lack of evidence-based treatment (18.8%). Fewer participants identified lack of motivation or low priority (15.6%) and time constraints (14.6%), accessibility barriers (8.3%) or other reasons (15.6%), while 10.4% reported no barriers.

**Table 5:** Barriers to providing non-cigarette tobacco cessation support.

Barrier	% (*n*)
**Lack of demand**	46.9 (45)
**Lack of training**	44.8 (43)
**Lack of knowledge**	43.8 (42)
**Lack of confidence**	20.8 (20)
**Language barriers**	19.8 (19)
**Lack of evidence-based treatment**	18.8 (18)
**Lack of motivation/low priority**	15.6 (15)
**Lack of time**	14.6 (14)
**Accessibility barriers**	8.3 (8)
**Other**	15.6 (15)
**None**	10.4 (10)

Among all participants (*n* = 96); participants were asked to select all that applied.

Open-ended responses provided further insight into structural and systemic challenges represented within the “other” category (Supplementary File 2). Funding and commissioning limitations emerged as the most prominent issue, mentioned by nine of the 15 participants who reported “other” barriers, with many noting that their services were not resourced or authorized to provide support for non-cigarette tobacco products. Some explicitly stated that “it’s not part of the funded programme” or “not in the stop smoking service specification.” A few participants also highlighted equity and access challenges, including low literacy among clients and difficulties reaching specific demographic groups—particularly older South Asian men—due to perceived cultural or language barriers.

### Training and resource needs

When asked about helpful training and resources, participants provided detailed open-ended feedback (Supplementary File 2). Training and education needs dominated responses, with many calling for bespoke, product-specific training on non-cigarette tobacco products—covering their composition, health risks, and cessation strategies. Participants repeatedly described gaps in existing NCSCT materials and requested additional or dedicated NCSCT modules focused on non-cigarette tobacco products, noting the need for “training and understanding of the product and how it is used” and asking whether “NRT and medication can be used to help quit” these products. Product knowledge gaps—such as unfamiliarity with how products were used, nicotine content, and harm profiles of heated tobacco, snuff, and chewing tobacco—were frequently highlighted, alongside uncertainty about how cessation approaches might differ from conventional cigarette support.

Participants also stressed the importance of accessible formats and delivery methods, with most favoring online modules, e-learning, and webinars for flexibility, while a smaller number preferred interactive face-to-face workshops, case studies, and peer reflection sessions. Practical, evidence-based resources were requested, including downloadable patient information leaflets, visual aids, and QR code-linked materials explaining product risks and available cessation options. One participant suggested “specific downloadable leaflets explaining risks of using, and examples of support options.”

Finally, several participants highlighted cultural and demographic considerations, particularly the need for multilingual materials and culturally competent services to reach groups where non-cigarette tobacco use is more prevalent. One comment noted the importance of “more multilingual specialists within the team.”

## Discussion

This survey of healthcare professionals involved in providing smoking cessation support in England examined awareness, confidence, training, and current practices regarding cessation support for non-cigarette tobacco products. Overall, our findings indicate that current service provision remains predominantly cigarette-focused, with limited preparedness to support users of alternative tobacco products. Awareness of more visible combustible products, such as cigars and shisha, was high; however, professional confidence and training lagged considerably, particularly for culturally specific and less visible products such as bidis and smokeless tobacco. Notably, shisha was often perceived by participants as less harmful than other combustible products, reflecting broader misconceptions about its risks despite evidence of significant toxicant exposure.^20^ These gaps signal a need for targeted workforce development, commissioning reform, and improved guidance to ensure cessation systems remain responsive to changing patterns of tobacco use.

A striking pattern was the disconnect between awareness, training, and confidence. While cigars, pipes, and shisha were almost universally recognized, fewer than one in five respondents reported receiving product-specific training, and training rates for bidis and moist snuff/dip were minimal. Despite this, over 40% felt very or extremely confident in supporting cigar cessation, with qualitative responses suggesting confidence often stemmed from the assumption that cigarette-based strategies generalize to all tobacco products rather than from specialist expertise. Qualitative responses described some attempts at product-specific adjustments (eg, emphasizing oral cancer risk for smokeless tobacco, addressing social aspects of shisha), but also highlighted uncertainty—particularly for emerging products such as heated tobacco (as has been documented in other countries^21^). Advisors also noted that some non-cigarette products are used non-daily and in social or cultural contexts. Although not explicitly stated, these patterns may present additional challenges for cessation support, as standard training tends to focus on daily cigarette use and framing tobacco use as inherently negative, rather than navigating situations in which use is culturally significant or socially normative.

These findings reflect the historical orientation of England’s tobacco control infrastructure. Stop smoking services were developed primarily to support cigarette cessation, offering behavioral support alongside pharmacotherapies such as NRT, varenicline, and bupropion.^22^ While this model is effective for cigarettes,^23^ and there is some evidence that certain treatments can help people to quit smokeless tobacco,^24^ the support offered by services may not adequately address the distinct use patterns and nicotine delivery profiles seen with non-cigarette products. For example, shisha and some smokeless tobacco products are often embedded in social or cultural contexts,^8–15^ while cigars and pipes involve different nicotine delivery profiles and patterns of use compared with cigarettes.^25^ Reliance on cigarette-based approaches in the absence of tailored evidence risks suboptimal outcomes and underscores the need for product-specific cessation strategies.

Equity considerations are central. Products disproportionately used by South Asian communities—such as bidis and certain smokeless tobacco products—had the lowest awareness, training, and reported support. In contrast, shisha, which is more prevalent,^2^ highly visible, and more strongly associated with Middle Eastern communities in England,^8–10^ was more familiar to participants but still lacked consistent tailored support. Without tailored guidance and proactive outreach, the current system risks widening tobacco-related disparities for groups with higher use of culturally specific products.

Training needs were repeatedly emphasized. Participants requested product-specific education and practical resources, including modules on composition, health risks, patterns of dependence, and effective cessation strategies. Digital and modular delivery formats were preferred, aligned with workforce needs and NCSCT delivery models.^26^ The emphasis on multilingual materials reflects wider evidence that culturally tailored cessation resources can improve engagement among minority ethnic groups and users of culturally specific tobacco products.^27^ Co-design with affected communities could help ensure that training resources and interventions are culturally appropriate, accepted by users, and aligned with their lived experiences.

Structural factors also constrained provision. Commissioning restrictions were widely reported, with services in some areas funded only for cigarette cessation, and in certain cases pharmacotherapies available only to cigarette smokers. In this context, limited practitioner knowledge of treatment options for non-cigarette tobacco products may reflect the absence of commissioned pathways, training opportunities, and clinical incentives, rather than individual knowledge deficits. These are barriers that are also reflected in qualitative work among health care professionals with experience in providing support for non-cigarette tobacco.^28^

Nearly half of respondents cited low demand as a barrier, yet this may reflect limited awareness of services or trust among affected communities to use services rather than lack of need. Practitioner-reported demand may also partly reflect the relative prevalence of different non-cigarette products in the population. For example, products used by only a small proportion of tobacco users, such as bidis or certain smokeless tobacco types,^2^ may generate fewer requests for support, even if individual users would benefit from intervention. Low observed demand should therefore not be interpreted as indicating a lack of population-level need. Explicit inclusion of non-cigarette products in commissioning frameworks, guidelines, and performance metrics will be essential to enable cessation service practitioners to apply new skills and resources in practice.

Inclusive and precise terminology is also important for both public health messaging and cessation service provision. Messaging that focuses only on “smoking” or “cigarettes” may not resonate with users of non-cigarette tobacco—particularly smokeless tobacco or waterpipe—leading to underreporting of tobacco use behaviors, misunderstanding of harms, and perceived or actual lack of cessation support. Likewise, practitioners may miss opportunities to provide support if routine assessments do not explicitly ask about non-cigarette tobacco products. Ensuring campaigns and service protocols explicitly encompass all forms of tobacco can help address gaps in awareness, perceived harms, and engagement with cessation support.^29^

Findings should be interpreted in light of the practitioner-level nature of the sample. Most respondents were individual healthcare professionals involved in smoking cessation support, rather than service managers or commissioners, and many had relatively limited time in role. This may have influenced reported awareness, confidence, and preparedness to support cessation for less commonly encountered non-cigarette tobacco products, particularly where services were not commissioned to provide such support. Importantly, practitioner-reported demand reflects what is observed within routine practice and may not fully capture service-level or population-level need. In particular, triage processes, commissioning arrangements, and organizational structures may shape which clients are seen by individual practitioners and which tobacco products are prioritized within services.

This study had several limitations. The convenience sample may over-represent healthcare professionals with greater interest in tobacco treatment. GPs (~1%) and pharmacists (0%) were substantially under-represented, limiting generalizability to primary-care and pharmacy settings. Survey respondents do not represent services across the United Kingdom and were disproportionately based in the South East of England. We did not capture or compare the ethnicity or demographic profiles of local populations, which may influence demand for cessation support for products more commonly used in specific communities. Awareness was low for some products, so sample sizes were small for analyses limited to those aware of these products. The cross-sectional design captures a single time-point and self-report measures may be subject to recall or social desirability bias and reflects perceived rather than actual service provision. Nonetheless, the study highlights systemic gaps in training, commissioning, and readiness to support non-cigarette tobacco users.

In conclusion, non-cigarette tobacco use in England is diverse and evolving, yet cessation support remains largely modeled on cigarette smoking. High confidence in the absence of product-specific training, coupled with low preparedness for culturally prevalent products, risks compromising support quality, and exacerbating inequalities. Strengthening provision will require coordinated action: commissioning reforms, product-specific clinical guidance, training aligned with national guidelines, and co-designed resources reflecting diverse use patterns and dependence profiles.

## Supplementary Material

ntag071_Supplemental_Files

## Data Availability

Data are available from the corresponding author on request.
