# Supplementary material for: Non-cigarette tobacco cessation support in England: current provision, confidence, and barriers identified in a cross-sectional survey of healthcare professionals involved in smoking cessation support
Source: Nicotine Tob Res. 2026 Apr 3;28(9):1529–38. doi: 10.1093/ntr/ntag071 (PMC13500351; doi:10.1093/ntr/ntag071)
Supplement: Supplementary file 1 [file ntag071_supplemental_files.zip › Supplementary_File_1_-_survey_measures_ntag071.pdf]

SECTION 1: PARTICIPANT CHARACTERISTICS

Q1. What is your job role?

0. Stop smoking / tobacco dependency advisor
1. GP
2. Pharmacist
3. Dental practitioner
4. Other (please specify job title and sector)

Q2. In what setting is your primary workplace?

0. Community
1. Hospital (outpatient)
2. Hospital (inpatient)
3. Other

Q3. Does your service/practice focus on a specific population group or groups (for example, young people, people who are pregnant, people from ethnic minority groups)?

0. No
1. Yes

Q3-1. If you choose 1. yes, Please specify which group or groups you focus on.

[Open-ended response box]

Q4. Does your service/practice have specific eligibility criteria for clients to receive support for tobacco/smoking cessation?

0. No
1. Yes

Q4-1.If you choose 1. yes, please specify the eligibility criteria.

[Open-ended response box]

This survey focuses specifically on services within **England**. If your workplace or service provision is located outside of England, please answer based on your primary location in England.

Q5. Where in England is your service/practice located?

0. North East
1. North West
2. Yorkshire and the Humber
3. West Midlands
4. East Midlands
5. East of England
6. London
7. South East
8. South West
9. Other

Q6. Please enter the first part of the postcode where your service/practice is located.

[Open-ended response box]

Q7. To the nearest year, how long have you personally been providing smoking cessation support?

[Open-ended response box]

Q8. What is the typical duration of tobacco/smoking cessation support that you personally provide?

0. Less than 4 weeks
1. 4-8 weeks
2. 9-12 weeks
3. More than 12 weeks

4. Don't know

Q9. How often do people approach your service looking for support to stop smoking cigarettes?

0. Daily

1. Less than daily but at least weekly

2. Less than weekly but at least monthly

3. Less than monthly but at least once a year

4. Less than once a year

5. Don't know

Q10. Which group do you consider you belong to?

1. Arab

2. Asian/Asian British - Bangladeshi

3. Asian/Asian British - Chinese

4. Asian/Asian British - Indian

5. Asian/Asian British - Pakistani

6. Asian/Asian British - Any other Asian background

7. Black - African

8. Black - Caribbean

9. Black - Any other Black / African / Caribbean background

10. Mixed - White and Asian

11. Mixed - White and Black African

12. Mixed - White and Black Caribbean

13. Mixed - Any other Mixed / multiple ethnic background

14. White - English / Welsh / Scottish / Northern Irish / British

15. White - Gypsy or Irish Traveller

16. White - Irish

17. White - Any other White background

18. Any other ethnic group

SECTION 2 - Cigars	

SECTION 2: AWARENESS AND SUPPORT PROVISION (NON-CIGARETTE TOBACCO)

Next, we want to ask you about nine different tobacco products. For each product, we will provide you with a description and ask the same set of questions.

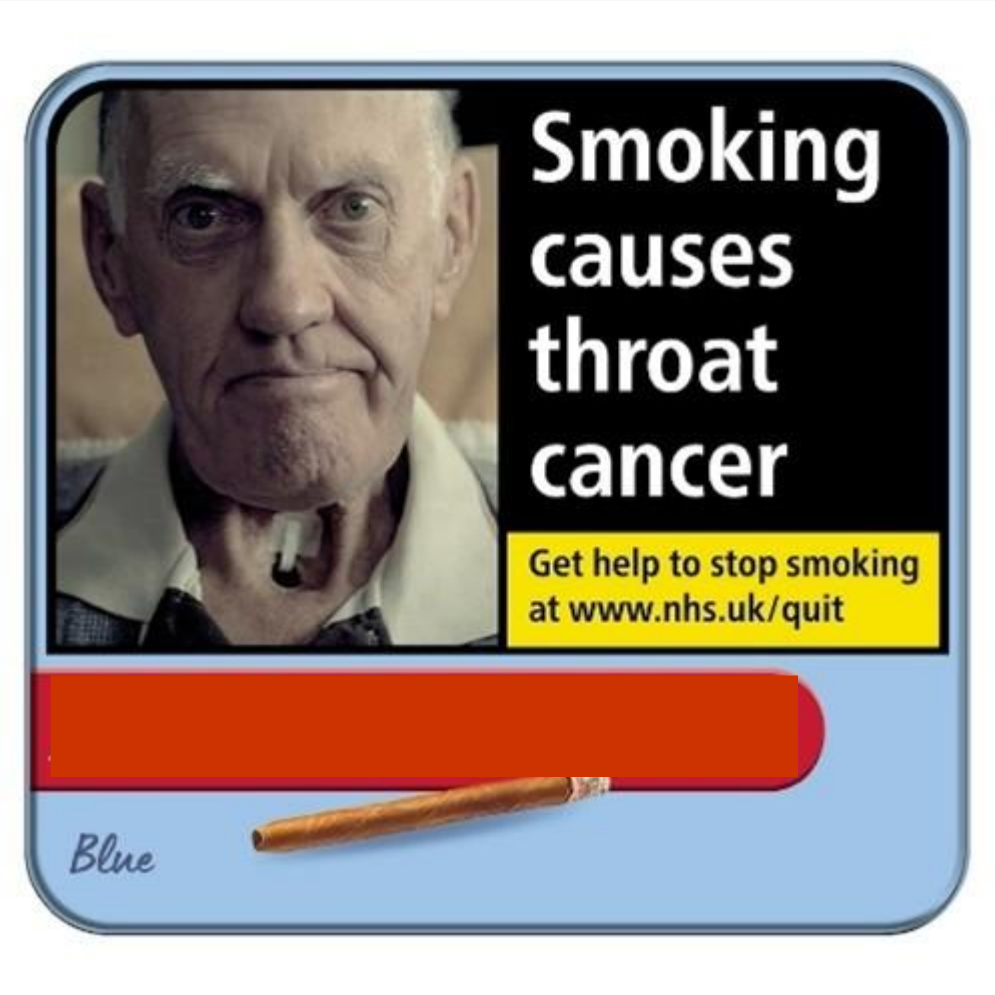

- Cigars: tobacco wrapped in tobacco leaf or paper that contains tobacco.

The next questions are about cigars.

Q11. Before this survey, had you seen or heard of cigars?

0. No
1. Yes
2. Don't know

Q12. How harmful do you think cigars are compared to regular cigarettes?

0. Not harmful at all
1. A lot less harmful
2. A little less harmful
3. About the same
4. A little more harmful
5. A lot more harmful
6. Don't know

Q13. Based on what you know or believe, does using cigars cause cancer?

0. No
1. Yes
2. Don't know

Q14. How often do people approach your service looking for support to stop smoking cigars?

0. Daily
1. Less than daily but at least weekly
2. Less than weekly but at least monthly
3. Less than monthly but at least once a year
4. Less than once a year
5. Don't know

Q15. Have you received any training in providing cessation support to people who smoke cigars?

0. No

1. Yes

2. Don't know

Q15-1. If yes, please describe the types of training you have received in providing cessation support to people who smoke cigars.

[Open-ended response box]

Q16. How confident do you feel in supporting people to stop smoking cigars?

0. Not at all confident

1. Not very confident

2. Somewhat confident

3. Very confident

4. Extremely confident

5. Don't know

Q17. Do you offer support to people who want to stop smoking cigars?

0. No

1. Yes

2. Don't know

Q17-1. What types of support do you currently offer to people who want to stop using cigars? Please select all that apply.

0. Behavioural support

1. Nicotine replacement therapy (e.g., gums, patches, lozenges, or sprays)

2. Varenicline (Champix)

3. Cytisine

4. Bupropion (Zyban)

5. Provision of e-cigarettes

6. Advice on e-cigarettes as a cessation aid

7. Advice on nicotine pouches as a cessation aid

8. Referral to other support

9. Other

Q18. Does the support you offer to people who want to stop smoking cigars differ from the support you offer to people who want to stop smoking cigarettes?

0. No

1. Yes

2. Don't know

Q18-1. If yes, please explain how the support you offer differs.

[Open-ended response box]

SECTION 2 - Cigarillos

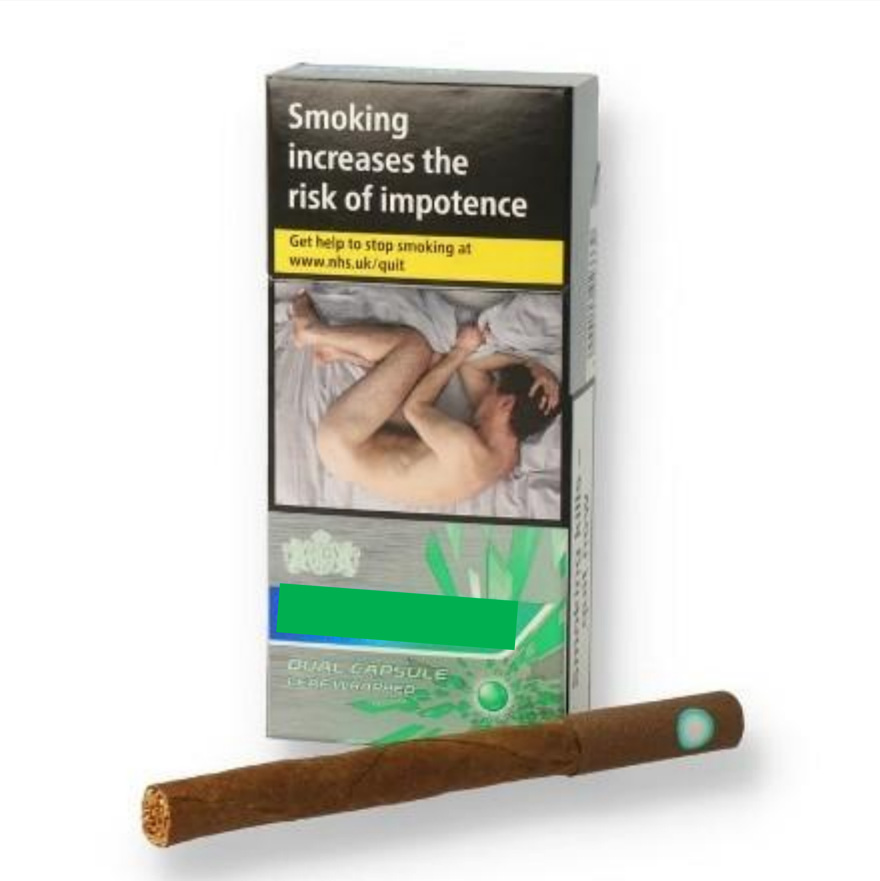

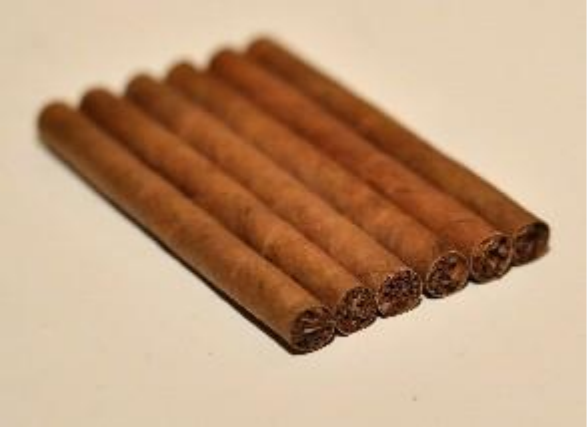

- Cigarillos: short, narrow cigars which usually have filters

The next questions are about cigarillos.

Q19. Before this survey, had you seen or heard of cigarillos?

0. No
1. Yes
2. Don't know

Q20. How harmful do you think cigarillos are compared to regular cigarettes?

0. Not harmful at all
1. A lot less harmful
2. A little less harmful
3. About the same
4. A little more harmful
5. A lot more harmful
6. Don't know

Q21. Based on what you know or believe, does smoking cigarillos cause cancer?

0. No
1. Yes
2. Don't know

Q22. How often do people approach your service looking for support to stop smoking cigarillos?

0. Daily
1. Less than daily but at least weekly
2. Less than weekly but at least monthly

3. Less than monthly but at least once a year

4. Less than once a year

5. Don't know

Q23. Have you received any training in providing cessation support to people who smoke cigarillos?

0. No

1. Yes

2. Don't know

Q23-1. If yes, please describe the types of training you have received in providing cessation support to people who smoke cigarillos.

[Open-ended response box]

Q24. How confident do you feel in supporting people to stop smoking cigarillos?

0. Not at all confident

1. Not very confident

2. Somewhat confident

3. Very confident

4. Extremely confident

5. Don't know

Q25. Do you offer support to people who want to stop smoking cigarillos?

0. No

1. Yes

2. Don't know

Q25-1. What types of support do you currently offer to people who want to stop smoking cigarillos? Please select all that apply.

0. Behavioural support
1. Nicotine replacement therapy (e.g., gums, patches, lozenges, or sprays)
2. Varenicline (Champix)
3. Cytisine
4. Bupropion (Zyban)
5. Provision of e-cigarettes
6. Advice on e-cigarettes as a cessation aid
7. Advice on nicotine pouches as a cessation aid
8. Referral to other support
9. Other

Q26. Does the support you offer to people who want to stop smoking cigarillos differ from the support you offer to people who want to stop smoking cigarettes?

0. No
1. Yes
2. Don't know

Q26-1. If yes, please explain how the support you offer differs.

[Open-ended response box]

SECTION 2 - Waterpipes

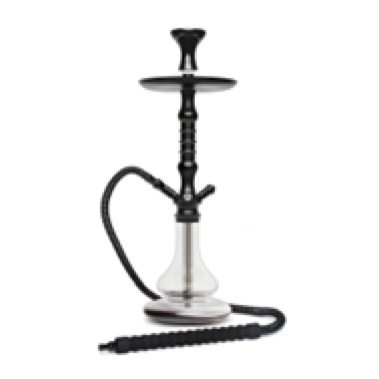

- Shisha: an instrument which draws tobacco smoke, often flavoured, through water for cooling and filtering before inhalation (also called waterpipes, hookah, nargile)

The next questions are about shisha.

27. Before this survey, had you seen or heard of shisha?

0. No
1. Yes
2. Don't know

Q28. How harmful do you think shisha are compared to regular cigarettes?

0. Not harmful at all
1. A lot less harmful
2. A little less harmful
3. About the same
4. A little more harmful
5. A lot more harmful
6. Don't know

Q29. Based on what you know or believe, does smoking shisha cause cancer?

0. No

1. Yes

2. Don't know

Q30. How often do people approach your service looking for support to stop smoking shisha?

0. Daily

1. Less than daily but at least weekly

2. Less than weekly but at least monthly

3. Less than monthly but at least once a year

4. Less than once a year

5. Don't know

Q31. Have you received any training in providing cessation support to people who smoke shisha?

0. No

1. Yes

2. Don't know

Q31-1. If yes, please describe the types of training you have received in providing cessation support to people who smoke shisha.

[Open-ended response box]

Q32. How confident do you feel in supporting people to stop smoking shisha?

0. Not at all confident

1. Not very confident

2. Somewhat confident

3. Very confident

4. Extremely confident

5. Don't know

Q33. Does your service offer support to people who want to stop smoking shisha?

0. No

1. Yes

2. Don't know

Q33-1. What types of support do you currently offer to people who want to stop smoking shisha? Please select all that apply.

0. Behavioural support

1. Nicotine replacement therapy (e.g., gums, patches, lozenges, or sprays)

2. Varenicline (Champix)

3. Cytisine

4. Bupropion (Zyban)

5. Provision of e-cigarettes

6. Advice on e-cigarettes as a cessation aid

7. Advice on nicotine pouches as a cessation aid

8. Referral to other support

9. Other

Q34. Does the support you offer to people who want to stop smoking shisha differ from the support you offer to people who want to stop smoking cigarettes?

0. No

1. Yes

2. Don't know

Q34-1. If yes, please explain how the support you offer differs.

[Open-ended response box]

SECTION 2 - Pipes

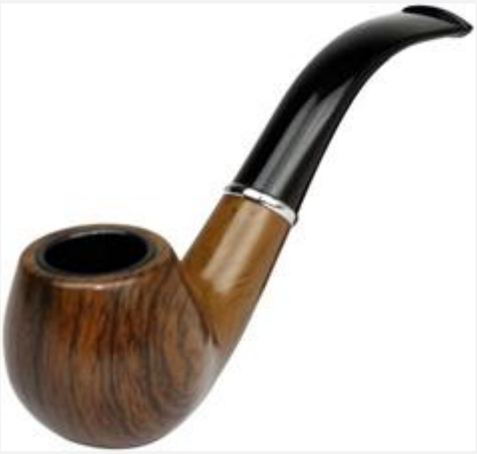

Pipes: device that holds loose tobacco which is lit and inhaled through a stem/mouthpiece

The next questions are about pipes.

Q35. Before this survey, had you seen or heard of pipes?

0. No
1. Yes
2. Don't know

Q36. How harmful do you think pipes are compared to regular cigarettes?

0. Not harmful at all
1. A lot less harmful
2. A little less harmful
3. About the same
4. A little more harmful
5. A lot more harmful
6. Don't know

Q37. Based on what you know or believe, does smoking pipes cause cancer?

0. No

1. Yes

2. Don't know

Q38. How often do people approach your service looking for support to stop smoking pipes?

0. Daily

1. Less than daily but at least weekly

2. Less than weekly but at least monthly

3. Less than monthly but at least once a year

4. Less than once a year

5. Don't know

Q39. Have you received any training in providing cessation support to people who smoke pipes?

0. No

1. Yes

2. Don't know

Q39-1. If yes, please describe the types of training you have received in providing cessation support to people who smoke pipes.

[Open-ended response box]

Q40. How confident do you feel in supporting people to stop smoking pipes?

0. Not at all confident

1. Not very confident

2. Somewhat confident

3. Very confident

4. Extremely confident

5. Don't know

Q41. Do you offer support to people who want to stop smoking pipes?

0. No

1. Yes

2. Don't know

Q41-1. What types of support do you currently offer to people who want to stop smoking pipes? Please select all that apply.

0. Behavioural support

1. Nicotine replacement therapy (e.g., gums, patches, lozenges, or sprays)

2. Varenicline (Champix)

3. Cytisine

4. Bupropion (Zyban)

5. Provision of e-cigarettes

6. Advice on e-cigarettes as a cessation aid

7. Advice on nicotine pouches as a cessation aid

8. Referral to other support

9. Other

Q42. Does the support you offer to people who want to stop smoking pipes differ from the support you offer to people who want to stop smoking cigarettes?

0. No

1. Yes

2. Don't know

Q42-1. If yes, please explain how the support you offer differs.

[Open-ended response box]

SECTION 2 - Bidis

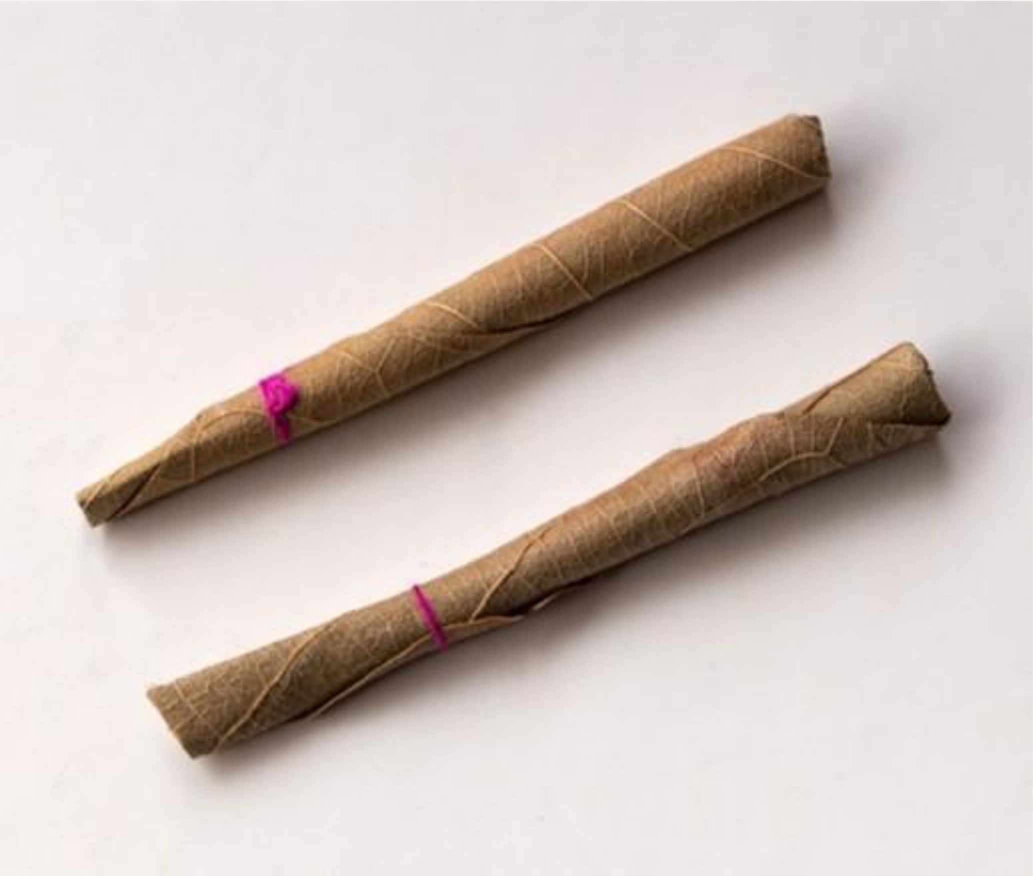

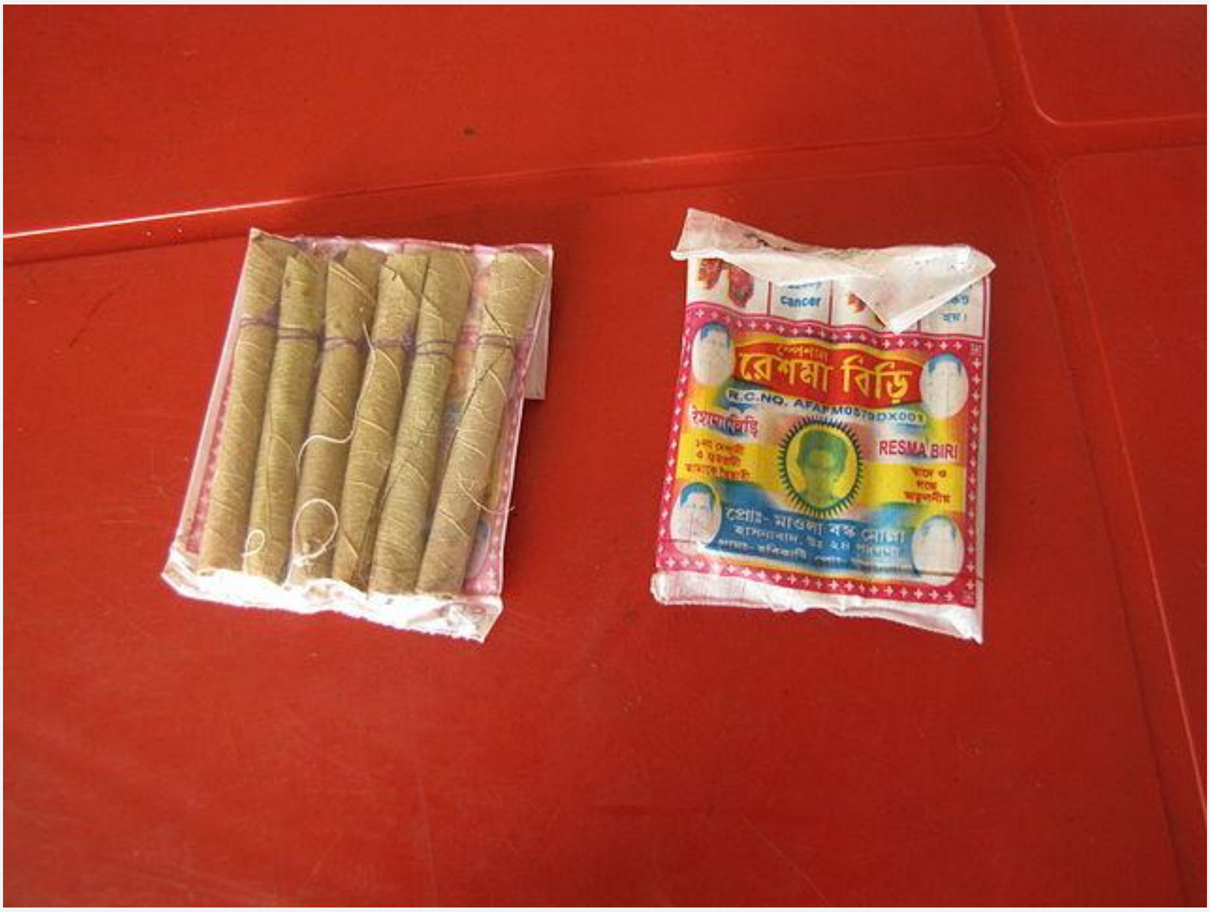

- **Bidis:** little cigarettes that are hand-rolled in tobacco leaves, tied with string at the ends, and may come in different flavours

The next questions are about bidis.

Q43. Before this survey, had you seen or heard of bidis?

0. No

1. Yes

2. Don't know

Q44. How harmful do you think bidis are compared to regular cigarettes?

0. Not harmful at all

1. A lot less harmful

2. A little less harmful

3. About the same

4. A little more harmful

5. A lot more harmful

6. Don't know

Q45. Based on what you know or believe, does smoking bidis cause cancer?

0. No

1. Yes

2. Don't know

Q46. How often do people approach your service looking for support to stop smoking bidis?

0. Daily

1. Less than daily but at least weekly

2. Less than weekly but at least monthly

3. Less than monthly but at least once a year

4. Less than once a year

5. Don't know

Q47. Have you received any training in providing cessation support to people who smoke bidis?

0. No

1. Yes

2. Don't know

Q47-1. If yes, please describe the types of training you have received in providing cessation support to people who smoke bidis.

[Open-ended response box]

Q48. How confident do you feel in supporting people to stop smoking bidis?

0. Not at all confident
1. Not very confident
2. Somewhat confident
3. Very confident
4. Extremely confident
5. Don't know

Q49. Do you offer support to people who want to stop smoking bidis?

0. No
1. Yes
2. Don't know

Q49-1. What types of support do you currently offer to people who want to stop smoking bidis? Please select all that apply.

0. Behavioural support
1. Nicotine replacement therapy (e.g., gums, patches, lozenges, or sprays)
2. Varenicline (Champix)
3. Cytisine
4. Bupropion (Zyban)
5. Provision of e-cigarettes
6. Advice on e-cigarettes as a cessation aid
7. Advice on nicotine pouches as a cessation aid

8. Referral to other support

9. Other

Q50. Does the support you offer to people who want to stop smoking bidis differ from the support you offer to people who want to stop smoking cigarettes?

0. No

1. Yes

2. Don't know

Q50-1. If yes, please explain how the support you offer differs.

[Open-ended response box]

SECTION 2 - Chewing tobacco

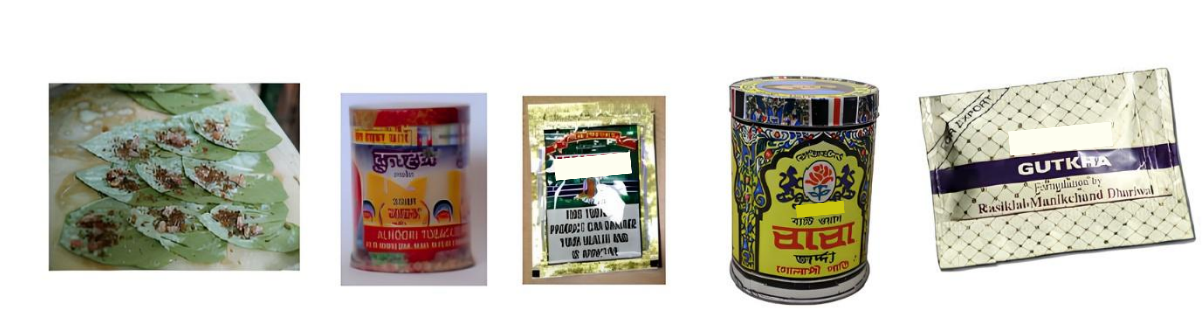

- Chewing tobacco: Available in different forms: dried whole and coarsely chopped tobacco leaves. Chewing tobacco can contain flavours such as clove or mint (e.g., zarda, gutka, gul, khaini, pan masala). Some forms are placed between the cheek and the lower gum. Some forms have to be chewed to release flavour and nicotine. Some chewing tobacco is consumed with areca nut and slaked lime, wrapped in betel quid (betel quid/ pan with tobacco).

The next questions are about chewing tobacco.

Q51. Before this survey, had you seen or heard of chewing tobacco?

0. No

1. Yes

2. Don't know

Q52. How harmful do you think chewing tobacco is compared to regular cigarettes?

(Please consider the types of chewing tobacco you most frequently observe or have general knowledge about)

0. Not harmful at all

1. A lot less harmful

2. A little less harmful

3. About the same

4. A little more harmful
5. A lot more harmful
6. Don't know

Q53. Based on what you know or believe, does chewing tobacco cause cancer?

0. No
1. Yes
2. Don't know

Q54. How often do people approach your service looking for support to stop chew tobacco?

0. Daily
1. Less than daily but at least weekly
2. Less than weekly but at least monthly
3. Less than monthly but at least once a year
4. Less than once a year
5. Don't know

Q54-1. What types of chewing tobacco do the people who approach your service for support use? Please select all that apply.

0. Zarda
1. Gutka
2. Gul
3. Khaini
4. Pan masala
5. Betel quid
6. Pan with tobacco

7. Naswar

8. Toombak

9. Other

10. Don't know

Q55. Have you received any training in providing cessation support to people who chew tobacco?

0. No

1. Yes

2. Don't know

Q55-1. If yes, please describe the types of training you have received in providing cessation support to people who chew tobacco.

[Open-ended response box]

Q56. How confident do you feel in supporting people to stop chew tobacco?

0. Not at all confident

1. Not very confident

2. Somewhat confident

3. Very confident

4. Extremely confident

5. Don't know

Q57. Does your service offer support to people who want to stop chew tobacco?

0. No

1. Yes

2. Don't know

Q57-1. What types of support do you currently offer to people who want to stop chew tobacco? Please select all that apply.

0. Behavioural support
1. Nicotine replacement therapy (e.g., gums, patches, lozenges, or sprays)
2. Varenicline (Champix)
3. Cytisine
4. Bupropion (Zyban)
5. Provision of e-cigarettes
6. Advice on e-cigarettes as a cessation aid
7. Advice on nicotine pouches as a cessation aid
8. Referral to other support
9. Other

Q58. Does the support you offer to people who want to stop chew tobacco differ from the support you offer to people who want to stop smoking cigarettes?

0. No
1. Yes
2. Don't know

Q58-1. If yes, please explain how the support you offer differs.

[Open-ended response box]

SECTION 2 - Dry snuff

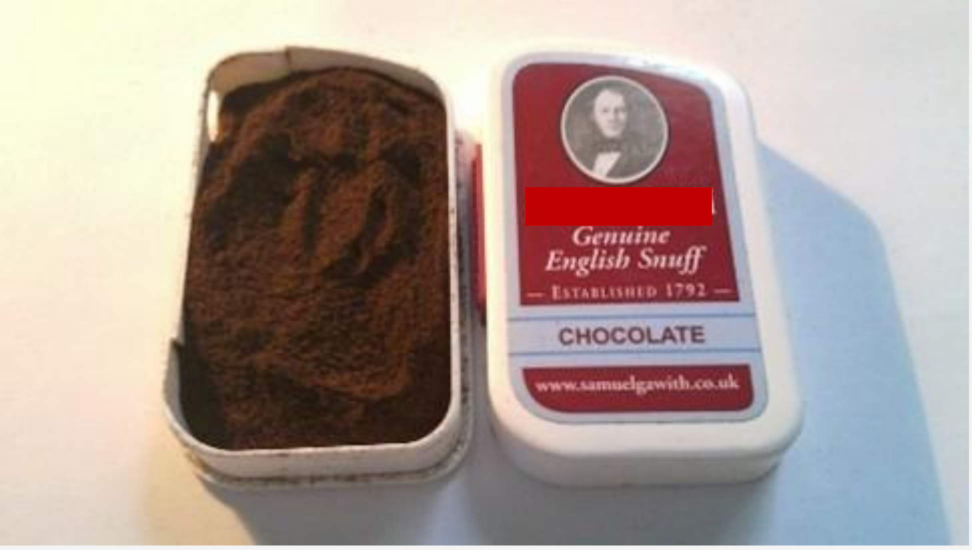

- Dry snuff: finely ground tobacco that is usually sniffed up the nose

The next questions are about snuff.

Q59. Before this survey, had you seen or heard of dry snuff?

0. No

1. Yes

2. Don't know

Q60. How harmful do you think dry snuff is compared to regular cigarettes?

0. Not harmful at all

1. A lot less harmful

2. A little less harmful

3. About the same

4. A little more harmful

5. A lot more harmful

6. Don't know

Q61. Based on what you know or believe, does using dry snuff cause cancer?

0. No

1. Yes

2. Don't know

Q62. How often do people approach your service looking for support to stop using dry snuff?

0. Daily

1. Less than daily but at least weekly

2. Less than weekly but at least monthly

3. Less than monthly but at least once a year

4. Less than once a year

5. Don't know

Q63. Have you received any training in providing cessation support to people who use dry snuff?

0. No

1. Yes

2. Don't know

Q63-1. If yes, please describe the types of training you have received in providing cessation support to people who smoke using dry snuff.

[Open-ended response box]

Q64. How confident do you feel in supporting people to stop using dry snuff?

0. Not at all confident

1. Not very confident

2. Somewhat confident

3. Very confident

4. Extremely confident

5. Don't know

Q65. Do you offer support to people who want to stop using dry snuff?

0. No

1. Yes

2. Don't know

Q65-1. What types of support do you currently offer to people who want to stop using dry snuff? Please select all that apply.

0. Behavioural support

1. Nicotine replacement therapy (e.g., gums, patches, lozenges, or sprays)

2. Varenicline (Champix)

3. Cytisine

4. Bupropion (Zyban)

5. Provision of e-cigarettes

6. Advice on e-cigarettes as a cessation aid

7. Advice on nicotine pouches as a cessation aid

8. Referral to other support

9. Other

Q66. Does the support you offer to people who want to stop using dry snuff differ from the support you offer to people who want to stop smoking cigarettes?

0. No

1. Yes

2. Don't know

Q66-1. If yes, please explain how the support you offer differs.

[Open-ended response box]

SECTION 2 - Moist snuff/dip

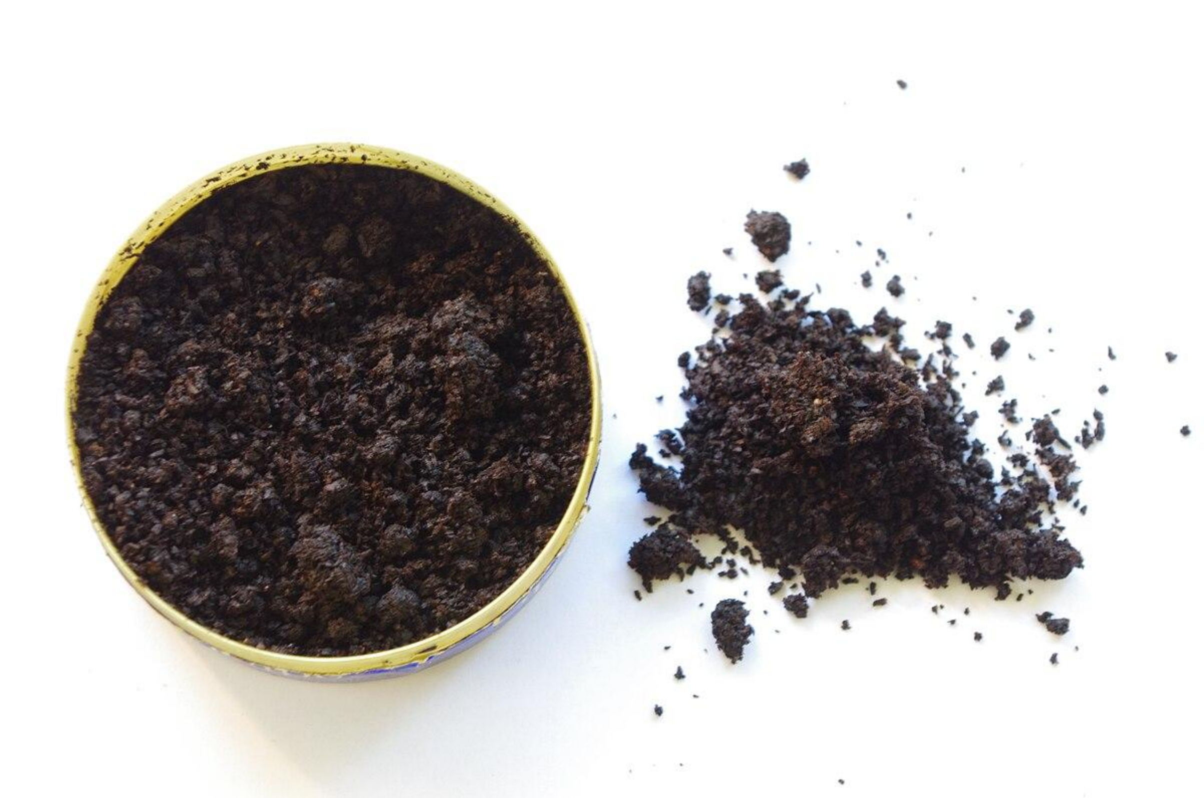

- **Moist snuff/dip:** finely ground tobacco that is usually placed between the gum and the lips. People who use dip often spit excess moisture out that accumulates during use.

The next questions are about snuff/dip.

Q67. Before this survey, had you seen or heard of moist snuff/dip?

0. No

1. Yes

2. Don't know

Q68. How harmful do you think moist snuff/dip is compared to regular cigarettes?

0. Not harmful at all

1. A lot less harmful

2. A little less harmful

3. About the same

4. A little more harmful

5. A lot more harmful

6. Don't know

Q69. Based on what you know or believe, does using moist snuff/dip cause cancer?

0. No

1. Yes

2. Don't know

Q70. How often do people approach your service looking for support to stop using moist snuff/dip?

0. Daily

1. Less than daily but at least weekly

2. Less than weekly but at least monthly

3. Less than monthly but at least once a year

4. Less than once a year

5. Don't know

Q71. Have you received any training in providing cessation support to people who use moist snuff/dip?

0. No

1. Yes

2. Don't know

Q71-1. If yes, please describe the types of training you have received in providing cessation support to people who use moist snuff/dip.

[Open-ended response box]

Q72. How confident do you feel in supporting people to stop using moist snuff/dip?

0. Not at all confident
1. Not very confident
2. Somewhat confident
3. Very confident
4. Extremely confident
5. Don't know

Q73. Do you offer support to people who want to stop using moist snuff/dip?

0. No
1. Yes
2. Don't know

Q73-1. What types of support do you currently offer to people who want to stop using dip? Please select all that apply.

0. Behavioural support
1. Nicotine replacement therapy (e.g., gums, patches, lozenges, or sprays)
2. Varenicline (Champix) /
3. Cytisine
4. Bupropion (Zyban)
5. Provision of e-cigarettes
6. Advice on e-cigarettes as a cessation aid
7. Advice on nicotine pouches as a cessation aid

8. Referral to other support

9. Other

Q74. Does the support you offer to people who want to stop using moist snuff/dip differ from the support you offer to people who want to stop smoking cigarettes?

0. No

1. Yes

2. Don't know

Q74-1. If yes, please explain how the support you offer differs.

[Open-ended response box]

SECTION 2 - Heated tobacco products

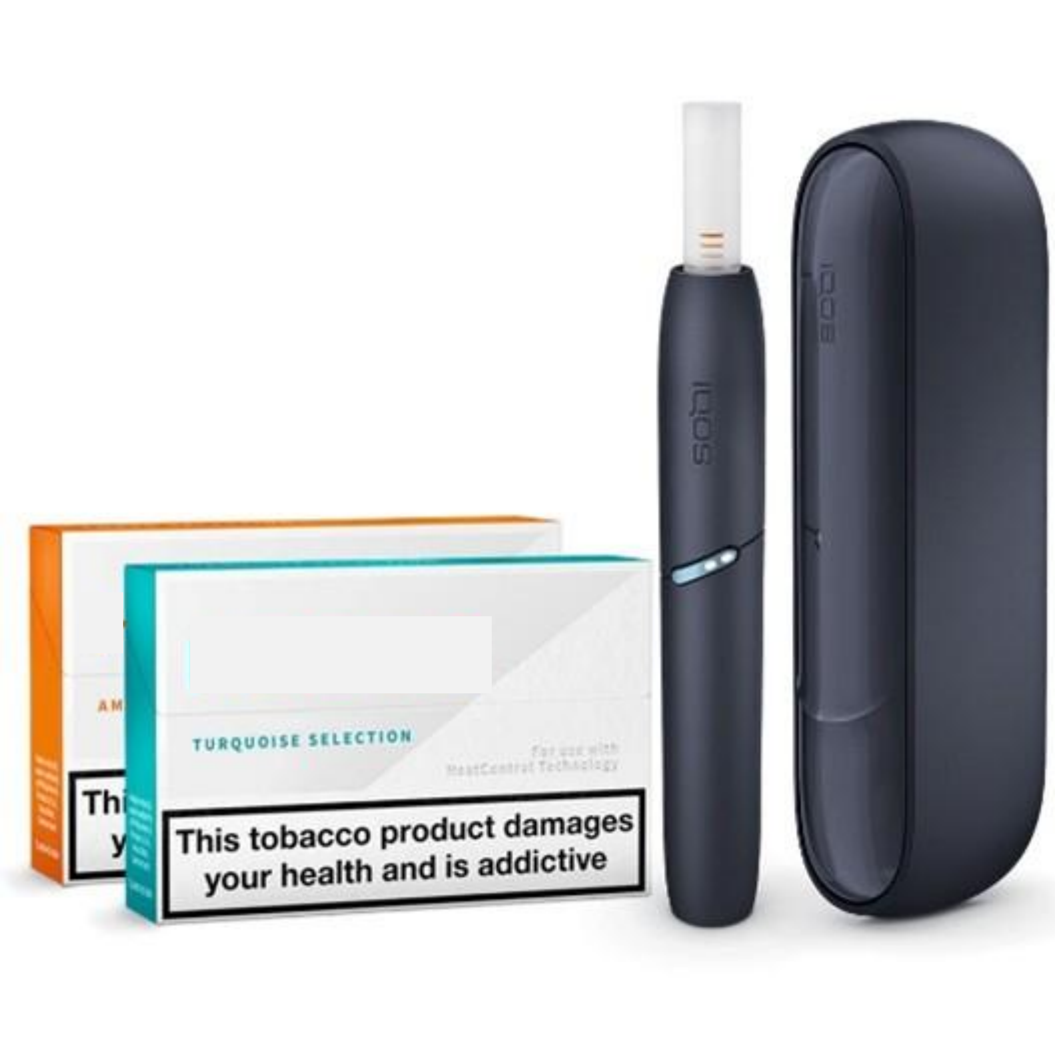

- Heated tobacco products: devices that heat tobacco (in sticks or capsules) to create an aerosol that is inhaled. These are NOT THE SAME as e-cigarettes, which use nicotine liquids and do not contain tobacco. Heated tobacco products include brands like IQOS/HEETS and Ploom.

The next questions are about heated tobacco.

Q75. Before this survey, had you seen or heard of heated tobacco products?

0. No

1. Yes

2. Don't know

Q76. How harmful do you think heated tobacco products are compared to regular cigarettes?

0. Not harmful at all

1. A lot less harmful

2. A little less harmful

3. About the same

4. A little more harmful

5. A lot more harmful

6. Don't know

Q77. Based on what you know or believe, does using heated tobacco products cause cancer?

0. No

1. Yes

2. Don't know

Q78. How often do people approach your service looking for support to stop using heated tobacco products?

0. Daily

1. Less than daily but at least weekly

2. Less than weekly but at least monthly

3. Less than monthly but at least once a year

4. Less than once a year

5. Don't know

Q79. Have you received any training in providing cessation support to people who use heated tobacco products?

0. No
1. Yes
2. Don't know

Q79-1. If yes, please describe the types of training you have received in providing cessation support to people who use heated tobacco products

[Open-ended response box]

Q80. How confident do you feel in supporting people to stop using heated tobacco products?

0. Not at all confident
1. Not very confident
2. Somewhat confident
3. Very confident
4. Extremely confident
5. Don't know

Q81. Do you offer support to people who want to stop using heated tobacco products?

0. No
1. Yes
2. Don't know

Q81-1. What types of support do you currently offer to people who want to stop using heated tobacco products? Please select all that apply.

0. Behavioural support
1. Nicotine replacement therapy (e.g., gums, patches, lozenges, or sprays)

2. Varenicline (Champix)

3. Cytisine

4. Bupropion (Zyban)

5. Provision of e-cigarettes

6. Advice on e-cigarettes as a cessation aid

7. Advice on nicotine pouches as a cessation aid

8. Referral to other support

9. Other

Q82. Does the support you offer to people who want to stop using heated tobacco products differ from the support you offer to people who want to stop smoking cigarettes?

0. No

1. Yes

2. Don't know

Q82-1. If yes, please explain how the support you offer differs.

[Open-ended response box]

SECTION 3: BARRIERS AND FACILITATIORS FOR PROVIDING EFFECTIVE SUPPORT (NON-CIGARETTE TOBACCO)

Q83. Which, if any, of the following do you think make it difficult for you or your service to provide support for cessation of non-cigarette tobacco products? Please select all that apply.

0. Lack of time

1. Lack of knowledge

2. Lack of confidence

3. Lack of training

4. Lack of demand

5. Lack of evidence-based treatment

6. Lack of motivation/low priority

7. Language barriers

8 . Accessibility barriers

9. Other

10. None

Q84. What specific training or resources do you think would be helpful to provide better support for people who want to stop using non-cigarette tobacco?

[Open-ended response box]

Q85. Are you aware of referral pathways for specialised cessation support for people who use non-cigarette tobacco in your area?

0. No

1. Yes

2. Don't know

Q85-1. If yes, please explain in detail

[Open-ended response box]
