# Supplementary material for: Non-cigarette tobacco cessation support in England: current provision, confidence, and barriers identified in a cross-sectional survey of healthcare professionals involved in smoking cessation support
Source: Nicotine Tob Res. 2026 Apr 3;28(9):1529–38. doi: 10.1093/ntr/ntag071 (PMC13500351; doi:10.1093/ntr/ntag071)
Supplement: Supplementary file 1 [file ntag071_supplemental_files.zip › Supplementary_File_2_-_open_responses_ntag071.pdf]

Have you received any training in providing cessation support to people who [use specific product]? If yes, please describe the types of training you have received.

Cigars

Built into NCSCT training - knowledge learnt from clients

I apply the same training as I received to help people quit cigarettes. I don't make a distinction between cigarettes and cigars; they both contain tobacco and cause nicotine addiction.

Locally provided training within LSSS

ncsct

NCSCT

NCSCT - Very Brief Advice on Smoking: practitioner assessment NCSCT - Assessment of Core Knowledge and Practice Skills

ncsct , advise from other collegues and previous manager

NCSCT standard treatment programme

NCSCT training

ncsct training.

NCSCT- i think this is covered under the information about different types of tobacco products

Received formal training in providing cessation support through the National Centre for Smoking Cessation and Training (NCSCT). I am a certified Stop Smoking Practitioner. Training included evidence-based approaches to supporting individuals who use various tobacco products, including cigars. It covered behavioral support strategies, the use of licensed stop smoking medications, and tailoring interventions to meet the specific needs of cigar smokers.

Same as smoking cigarettes training

Smoking Cessation NCSCT training module

treated the same as people who smoke unfiltered cigarettes although need to remember that cigars are generally not smoked as often

Cigarillos

Locally trained within service

NCSCT

NCSCT - Very Brief Advice on Smoking: practitioner assessment NCSCT - Assessment of Core Knowledge and Practice Skills

NCSCT standard treatment programme

NCSCT training.

Same as cigarette training

Shisha

A little about Shisha awareness

A minimal amount in the standard treatment programme through NCSCT

general advice and support.

information on use amongst different demogarphic groups, how it's used and impact

Locally

Mentioned briefly in NCSCT training

NCSCT

NCSCT - smoking other Tobacco products

NCSCT - Very Brief Advice on Smoking: practitioner assessment NCSCT - Assessment of Core Knowledge and Practice Skills

ncsct . previous manager UKNSCC conferences in the past

NCSCT and in-house training

NCSCT training

NCSCT training and inhouse training.

NCSCT- smoking other tobacco products

NSCSCT module

Through NCSC Training on effects and prevalence of shisha inhalation.

We had an information sharing session with HAREF - Haref is a network dedicated to improving health and wellbeing for ethnically minoritized communities in Newcastle and Gateshead

Pipes

ncsct

NCSCT

NCSCT - other tobacco products

NCSCT - Very Brief Advice on Smoking: practitioner assessment NCSCT - Assessment of Core Knowledge and Practice Skills

NCSCT and in house training

NCSCT Standard treatment programme

NCSCT training. None of the Clients Il have helped quit nicotine addiction over 5 years have smoked a pipe.

NCSCT- other tobacco products

NCST Training on awareness of ham and effects of pipe tobacco smoking.

nctsct

Same a we would offer any one that smokes cigarettes and cigars

treat as a cigarette smoker ,still inhaling burning tobacco therfore risks are the same, also pipe tends to be held in the mouth most of the time .

Bidis

Awareness on what Bidi is.

general info and use by different groups

NCSCT

NCSCT - Very Brief Advice on Smoking: practitioner assessment NCSCT - Assessment of Core Knowledge and Practice Skills

ncsct and previous UKNSCC conferences

We had a workshop on different smoking habits and products that were more common in some Asian communities.

Chewing tobacco

A workshop - see previous answer.

As part of general training.

Awareness on bidi.

general information re use and impact

NCSCT

NCSCT - other tobacco products

NCSCT and in house training

NCSCT training.

NCSCT-other tobacco products

same as for cigarette

Very limited via NCSCT due to demographics of our area we do not encounter these products being used

Dry snuff

Awareness on what snuff is

in house training

NCSCT / ASH website guidance

Very limited

Moist snuff/dip

in house

Very limited

Heated tobacco

A little awareness on what they are and how they work

about the product and what it is

In house

information sheet

Local training

NCSCT

NCSCT - other tobacco products

NCSCT standard treatment programme

NCSCT training

NCSCT-

NCST Training on tobacco products

training received from Improving Performance in Practice Team on Tobacco dependence.

Does the support you offer to people who want to stop vaping differ from the support you offer to people who want to stop smoking cigarettes? If yes, please explain how the support you offer differs.

Cigars

All support is tailored to meet individual need, but all based on NCSCT standard treatment protocol

We'd discuss how and why they use cigars and the barriers to quitting. The association with cigars and it not being tobacco and the use in celebrations etc

Cigarillos

As before, we would discuss the use, barriers, reason for choice and nicotine dependence as well as treatment options.

similar but more emphasis on co gas and tar

Shisha

As before, we would talk about when, how and how often. Whether there is a social aspect. Eg. At Shisha cafe etc on holiday etc. As well as barriers to quitting.

general differences...co advice....use and age etc

We do not offer NRT/ Vapes for Shisha use. We would offer behavioural support for users, alongside relevant info on dangers of shisha and harm reduction on BBV.'s.

Pipes

As before, we'd discuss the use, choice or use and barriers to quitting which are unique to the person and the product or device they use.

Normally these people are not doing it every day but occasionally and socially

Bidis

[No responses]

Chewing tobacco

As before, I want to understand their use, dependence and unique circumstances around the use of chewing tobacco.

Behavioural support only

not able to provide certain products

these clients see one particular advisor . they cannot be included in SS returns whilst respiratory cancer is negated , mouth cancers and collapse it the issue with this form of tobacco product

We as a stop smoking service are NOT commissioned to provide support for chewing tobacco users only combustible tobacco. However, due to the population demand, we do offer behavioural support and NRT for these service users. The NRT gum is the most preferred product of service user choice as it replicates the chewing tobacco sensation.

Dry snuff

As before. I am keen to learn how, why, when they use it.

Behavioural support only

Moist snuff/dip

Behavioural support only

Heated tobacco

As before.

As IQOS or heated tobacco products are not as harmless as vapes we do offer this option to users who have been using them.

Which, if any, of the following do you think make it difficult for you or your service to provide support for cessation of non-cigarette tobacco products? Please select all that apply. If you choose 9. Other, please specify:

It is not included in our funding so budget wise we dont have for this type of thing

It's not a part of the funded programme

Literacy levels of users.

Newly re launching the service, I think the service is planning to further develop

Not commissioned

not commissioned to support these clients

Not funded to provide this

Not in the stop smoking service specification,

Our funding provider stipulates the budget is for lit/combusted tobacco only. Another section of the Service that I work for ie Social Prescribing (separately funded), can help with behaviour change for vapers, but generally they don't feel confident with this and they can't offer Nicotine Replacement Therapy. Also they don't have training for most of the products you mention but they do have access to the NCSCT training on helping people quit vaping and they have all had health coaching training and training in behaviour change support (as do the Stop Smoking Service Staff).

people dont request this...its all aimed at cigs and targets

people say they want to give up smoking but when contacted, they are not keen.

Restrictions on providing NRT/ Vapes to non combustible tobacco users by the LA.

Some contracts do not include non cig tobacco products in support.

There can be barriers to reaching BAME populations (usually older generation South Asian men) and raising awareness of our services.

This is not a service we provide. Its for people who smoke or Vape.

What specific training or resources do you think would be helpful to provide better support for people who want to stop using non-cigarette tobacco?

A Crash course on what they are, can NRT be used to support and what behavioural support can be provided

More multi-lingual , specialists within the team.

1. Bespoke training for non-cigarette tobacco products (similar to the provision for cigarettes) 2. Workshops, interactive role-play sessions, case studies, sharing best practice, peer reflection sessions, etc.

A better understanding of products they using and support we can provide

A better understanding of risks and long term effects

A generic non-cigarette video training containing products information and specific instructions

A training workshop and slides. Leaflets and website info.

Access to medications that help

Access to training modules on NSCST or available links to bring up the relevant info if service users needed specify info.

Additional modules on NCSCT training

an online resource to go over things

Any

Any training to educate and train people involved in delivering treatment and support.

Any training would be good either online or otherwise

Any training. I haven't seen any

As it's all fairly rare, I find doing my own research at the time into the products is helpful. NCSCT modules are great too.

Awareness in if support can be given via smoking cessation service if not how we can give information to help them and awareness.

basic knowledge and training on the different types of products available

Because I cover maternity with patients Antenatally through to Postnatally most are cigarette / roll up smokers and a few Cannabis so do date haven't really had much experience with other tobacco related products that were mentioned in this survey. Although it would be beneficial to have more product knowledge of the alternative smoking products available.

Better understanding of the support they can receive would encourage more people to try

Better understanding of what those products are. Whilst I have heard of them, I'm not entirely familiar within our current training whether there is great detail into those products. We also rarely/never see clients wanting to stop those products and I wonder whether it's two-fold; lack of knowledge on the clients behalf of the causes of cancer and these products effects, and/or a lack of advertising in smoking cessation campaigns, both nationally and locally, to promote the cessation of them (We hear smoking cessation and assume 'Cigarette smoking', perhaps services could be changed to 'Tobacco cessation').

Easily accessible visual resources and communications

Education over what taking tobacco in other forms can do to the body for example chewing tobacco. Education around shisha and how it makes it smother and easy to inhale and is not 'safe' as perceived.

evidence based research and less media scare messages. A lot of time is spent on correcting information and reassuring clients about what is actually harmful.

Face to face or at least teams with take away info to look back at

Familiarisation with the products and ways to best support clients

general training to give more knowledge on these products

help with being able to stop vaping

how best to offer the service - and best product to help client

I am not sure if there is training around this and I would not know where to look to find courses.

I have never been approached for support from patients wanting to quit products other than cigarettes and cigars

I think the training and resources offered are enough.

I think there is enough training available, but any info must be available in different languages for use within services

I would learn a lot from more experience working with people who want to quit non-cigarette tobacco products, s from case-studies. NSCST offers training but for me, I value learning from experiences with clients.

I would like evidence based training on heated tobacco products.

ICB need to commission stop smoking services to include in service spec.

including non smoking tobacco to the same range as smoking tobacco and not differentiating between the two- instead refer to as tobacco use

including Non-Cigarette training session in NCSCT

Information about all the different types, their strengths, their harmfulness to health, how to effectively support someone to quit.

Information leaflets QR code readers providing information around the dangers of these products

information re use, impact, evidence base for SS aids

introduce all these aforementioned products into our practitioner training programme and produce posters and leaflets to advertise the service potential new clients who are unaware that we can help them too.

it would be good to get more real stories and overviews of usage

leaflets

Leaflets E learning Courses

More face to face training and resources

More funding so we can provide NRT products for longer

More information about the non- cigarettes support use and evidence based treatment approach. Online platform where professionals from different Trust can share their views and learn from each other. Leaflets on non cigarette tobacco use that is patient friendly.

more information leaflets to discuss the risks

More information sharing on how they are used and nicotine levels etc to best support the person

more online information/ specific module on NCSCT

more products would help.

more training

More training and resources on snuff or how to confidently answer myths around smokeless tobacco

more training on all of the non cigarette tobacco products

More training on the specific non cigarette to tobacco products.

More videos and lectures and also guides which could be handy .

More visual aids

Most services focus on cigarette or rolling tobacco only. They don't train advisors in any other tobacco usage.

N/A

NCSacT online training maybe via Zoom/Teams/Webinar

NCSCT module. Case studies of clients who have experience using non-cigarette tobacco

NCSCT training

NCSCT training module or more information for advisors as i dont think we have the knowledge to deal with this correctly at the moment

NCSCT training platform

Nicotine Replacement Therapy and specific training for the different types of non-cigarette tobacco.

Non cigarette tobacco types and treatments

on line via NCSCT

online training

online training courses or manuals to refer to

Re training on champix etc

Resources to refer clients to the correct service

Separate dedicated and detailed training in NCSCT would help.

Similar training to that provided by NCSCT, standard treatment plans, what treatment is appropriate to the level of non cigarette products used.

some leaflets - but not a commissioned service in our area

Some of the above that I said i didnt know to much about e.g Sniff, pipe, chewing tobacco

Specialist training

Specific download able leaflets explaining risks of using, and examples of support options and how these can support quitting.

Specific NCSCT units for training on these topic. With video led learning. Webinars etc.

Specific NSCST guidelines More training on specific types of non-cigarette tobacco More research and evidence based treatments

Specific training for each non-cigarette tobacco item.

Specific training on the best quit aids for those products

this group tends to be older asian community. other younger newer less experienced staff may not have heard of some the products mentioned.   NB regarding Champix/ Varenicline. it has been withdrawn for around 2-3 years now, otherwise the boxes would have been ticked as and option! there are comunications coming through that this will soon be available again but we are still waiting for the go ahead to re commence providing it!

To provide better support for people who want to stop using non-cigarette tobacco products (such as cigars, shisha, or smokeless tobacco), it would be helpful to have more specific training and resources focused on these forms of tobacco. This could include: Tailored behavioural intervention techniques for different types of tobacco users. Updated evidence-based guidance on the health risks and addiction patterns associated with non-cigarette tobacco. Case studies and role-play scenarios involving users of cigars, shisha, or chewing tobacco. Cultural competence training, since the use of certain non-cigarette tobacco products may be more common in specific communities. Resources for multilingual and community-specific outreach, including leaflets, videos, and digital tools. Training on pharmacotherapy options and how they apply to users of non-cigarette tobacco products. These would enhance confidence and effectiveness in supporting a broader range of tobacco users.

Training and understanding of the product and how it is used. Is it the same behavioural support as quitting a cigarette. Can NRT and medication be used to help quit (guess so if it is tobacco)

Training is needed around all sorts of non-cigarette tobacco available, like how it works, how harmful it is and does NRT help to stop them. Also training on health professionals on how it looks like so they can know all about it and so can support their clients.

Training on how they are used and how the habits differ from smoking cigarettes

Training provided in our yearly refresher.

Training specially tailored around evidence based treatment for smokeless tobacco products. Funding resources for hospital based tobacco dependency service to support people who want to stop using non-cigarette tobacco.

Training webinar, information leaflets, online training

understanding the strength of non-cigarette tobacco and how to quantify use to provide the best form of nicotine replacement or medication. Also how to monitor quit levels.

We would support anyone as per our service guidelines- but would ensure we use NCSCT resources beforehand to update knowledge of specific products

Which NRT products would be most suited

Wider training on non-cigarette nicotine dependence.

Would be good to have information regarding the different products how they are used and what support we could offer

What types of support do you currently offer to people who want to stop [using product]? Please select all that apply. If you choose 8. Referral to other support, please specify:

Cigars

Community Referrals and Local Borough Referrals

Community Stop Smoking Service

Hypnotherapy On-line/ Apps for motivational support

If face to face is needed we refer

LA SSS

Local clinic & pharmacy

local sss

Local stop smoking services

Once discharged from our service a referral to a Local Authority Stop Smoking Service is offered

One You Lincolnshire (primary work area support -in community) also can reffer to other local community based on PT location

Pharmacy and community support on discharge from hospital

Referral to community SSS on discharge

Referral to the community stop smoking team

worcestershire public health service 'swap to stop' and 'smokefree homes'

Cigarillos

Community Stop Smoking Service

Face to face if we believe needed

local SSS

On Discharge

We do a 12 week programme and also referrer them to community SSS for follow up in community along side

When discharged from our service a referral to a local authority stop smoking service is offered

Worecestershire Public Health ,swap to stop' and 'smoke free homes'

Shisha

[No responses]

Pipes

can refer to community service

community SSS

Community Stop Smoking Service

F2F if needed

local SSS

on discharge

Pharmacy and community support on discharge from hospital

referral to community team

When discharged from our service a referral to a local authority stop smoking service is offered

Worcestershire Public Health 'swap to stop' and 'smoke free homes'

Bidis
community referral
Community Referrals
Community Stop Smoking Service
local SSS
Refer to LSSS
Worcestershire Public Health 'swap to stop' and 'smoke free homes'

Chewing tobacco
Community referrals
Community Stop Smoking Service Dental/Oral Health Practitioners
Local stop smoking services
We offer patient's a referral to the local SSS on discharge
When discharged from our service a referral to a local authority stop smoking service is offered
Worcestershire Public Health 'swap to stop' and 'smoke free homes'

Dry snuff
We offer patient's a referral to the local SSS on discharge
When discharged from our service a referral to a local authority stop smoking service is offered
Worcestershire Public Health 'swap to stop' and 'smoke free homes'

Moist snuff/dip
Community Stop Smoking Service - ones who provide support for smokeless tobacco products Dental/Oral Health Practitioners
When discharged from our service a referral to a local authority stop smoking service is offered
Worcestershire Public Health 'swap to stop' and 'smoke free homes'

Heated tobacco
Community Stop Smoking Service
LSSS
Pharmacy and community support on discharge from hospital
We offer patient's a referral to the local SSS on discharge
When discharged from our service a referral to a local authority stop smoking service is offered
Worcestershire Public Health 'swap to stop' and 'smoke free homes'

Are you aware of referral pathways for specialised cessation support for people who use non-cigarette tobacco in your area? If yes, please explain in detail.

Addiction rehab centres.

As explained above: I will copy and paste. Another section of the Service that I work for ie Social Prescribing (separately funded), can help with behaviour change for vapers, but generally they don't feel confident with this and they can't offer Nicotine Replacement Therapy. Also they don't have training for most of the products you mention but they do have access to the NCSCT training on helping people quit vaping and they have all had health coaching training and training in behaviour change support (as do the Stop Smoking Service Staff).

Brent Stop Tobacco service offers 6 week behavioural support and NRT for chewing tobacco and shisha users

Clients can self-refer through the website, QR code and telephone or be referred by a professional.

Community Stop Smoking Service - for smoke form non-cigarette tobacco users, sometimes smokeless form Dental Practitioners - for smokeless tobacco users

Currently, we refer patients to our community team if we come across patients who uses chewing tobacco or cigars

Depends on the area - not all have this

I guess LA SSS would help. So we generally refer to LA

I often have one you referrals

I would ask my manager.

If there is a part of the service we cannot provide then we refer back to One You Lincolnshire, who is our lead.

In our Borough if some one is Vaping we can assess them and the dosage of the Vape and support them to quit the Vape by reducing the dosage and behavioural support.

It would be to the local stop smoking service

Lincolnshire Recovery Partnership I think

local SSS

Multiple referral pathways to our service from primary and secondary care, partners and many other stakeholders

One You Lincolnshire (County-wide NHS-funded service) Offers a 12-week quit programme supporting all tobacco users, including non-cigarette forms.   Provides face-to-face, telephone, or online support, plus free Nicotine Replacement Therapy (patches, gum, lozenges-or vapes)

Our selves if pregnant or live well Wolverhampton for families and friends and non pregnant.

ourselves if pregnant or Live well Wolverhampton for family/ friends and non pregnant people

Refer to stop smoking service

same for all tobacco product

see previous stated information

They would be seen by our service

We do support if smoking tobacco - I haven't personally been approached by any on here other than the heated tobacco and at that time we couldn't. I would have to ask but I guess we could support with some of the above

we pass these clients to a particular member of staff. she records them on a spread sheet . there doesnt seem many lately in this area.

We refer patients to their local stop smoking services.

we refer to all local SSS whenever nwcessary

Worcestershire Public Health 'swap to stop' and 'smoke free homes'. Self referral available, but can also refer individuals with their consent.
